# Accelerating cardiovascular digital twin creation using precomputed virtual populations as Bayesian priors

**DOI:** 10.3389/fphys.2026.1950271

**Published:** 2026-09-21

**Authors:** Iris L. Shelly, Jon N. Peterson, Giulia Cornali, Jennifer Rohrs, Patrick E. Gelbach, Joe Alexander

**Affiliations:** 1 Medical and Health Informatics Laboratories, NTT Research, Inc., Sunnyvale, CA, United States; 2 Embody Biosciences, Corte Madera, CA, United States

**Keywords:** Bayesian inference, cardiovascular hemodynamics, digital twins, lumped parameter models, mechanistic models, parameter estimation, virtual populations

## Abstract

**Introduction:**

Mechanistic lumped-parameter models of the cardiovascular system can provide insight into underlying physiological processes that cannot be measured directly. When tailored to an individual patient, these models have the potential to inform treatment for conditions such as heart failure. We aim to develop a robust and repeatable method of constructing a virtual population, defined as a collection of parameter sets that spans the full range of expected phenotypic variability of a population of interest. Such virtual populations can then accelerate patient-specific Bayesian inference by serving as a precomputed prior distribution to determine probabilistic cardiovascular digital twins.

**Methods:**

This work presents a Bayesian approach to establishing a virtual population based on population-level measurement data. It utilizes the DiffeRential Evolution Adaptive Metropolis (DREAM) algorithm to approximate a Bayesian posterior distribution consistent with the published clinical data of healthy populations (19 pressure and volume measurements across samples of at least 125 patients apiece). It subsequently applies weight-based allometric scaling of the resulting posterior parameter sets to more fully match target measurement variability. We applied this technique to a mechanistic hemodynamic model beginning with 23 parameters of interest. The final virtual population is then used to rapidly compute 17 proof-of-concept patient-specific digital twins, using patient data from an independent cohort not considered during virtual population development.

**Results:**

Our methodology effectively replicated at least 74.6% of the expected coverage of the target measurement distributions, with 17 of 19 measurements being represented at more than 80% of the expected coverage. Seventeen patient-specific digital twins were then computed from within this virtual population, with mean absolute percentage error across measurements for each patient ranging from 0.71% to 8.61% relative to the clinical measurement targets and a mean error across patients of less than 3%.

**Conclusion:**

The proposed methodology represents a promising approach to developing a virtual population and demonstrates the feasibility of rapid posterior reweighting within such a precomputed virtual population, according to individual patient data. This computational proof-of-concept may help set the stage for future scalable creation of patient-specific digital twins to aid in clinical decision-making.

## 1 Introduction

Precision cardiology aims to tailor therapies to individual patients to improve the quality of care at an individual level while also increasing efficiency of the healthcare system as a whole (Corral-Acero et al., 2020; Akbarialiabad et al., 2024; Barricelli et al., 2019). Computational models can help to advance precision cardiology by simulating the biological processes underlying cardiovascular diseases and their associated comorbidities (Peirlinck et al., 2021; Kadem et al., 2023; Coorey et al., 2022; Thangaraj et al., 2024; Hose et al., 2019). Such a computational model tuned to a specific patient can then comprise the patient’s digital twin: a virtual representation of the individual patient that dynamically incorporates data and observations from the physical patient, helping clinicians to improve patient outcomes by more accurately predicting an individual’s response to treatments (Corral-Acero et al., 2020; Coorey et al., 2022; Sel et al., 2024; Bhagirath et al., 2024).

One promising computational basis for cardiovascular digital twins is a lumped-parameter mechanistic model, in which mathematical relationships are used to describe underlying physiological mechanisms, grouped at the level of topological compartments rather than spatial dimensions (Colebank et al., 2024; Doshi and Burkhoff, 2016; Kadem et al., 2023; Shi et al., 2011). However, despite the relative simplicity of these models compared to higher-dimensional, multi-scale modeling approaches, clinical usage is still not widespread, in part due to the difficulty of identifying appropriate patient-specific values for model parameters (Shimizu et al., 2018; Garber et al., 2022; Niederer et al., 2020). While model parameters are generally not measurable, the resulting simulation outputs often can be measured in clinical practice – for example, pressures and volumes in the context of a hemodynamic model (Colebank et al., 2024; Shimizu et al., 2018). Therefore, to facilitate clinical usage of these models, there is a need to tune parameter values to produce outputs consistent with patient-specific clinical measurements.

Bayesian inference, a mathematics of learning from evidence, is well-suited to this parameter estimation problem as it directly quantifies uncertainty, which is critical for safe and effective clinical applications (National Academies of Sciences, Engineering, and Medicine, 2024; Colebank et al., 2024; Seoni et al., 2023). Bayesian inference begins with a probability distribution describing knowledge uncertainty (the prior distribution), then every new piece of information reshapes that uncertainty distribution into a new one (the posterior distribution). In this framework, the prior distribution is the uncertainty of prior knowledge about parameters (from initial expectations or beliefs) before observing any measurement data (Colebank et al., 2024). The posterior distribution of parameters (Equation 1) is estimated by combining the prior distribution and a likelihood function:

(1) Posterior∝Prior·Likelihood

The likelihood function provides a probabilistic formulation of how plausible the observed measurement data are under the model given any set of parameters (Linden et al., 2022). Bayesian inference then computes the posterior distribution, which is the estimated probability distribution of the parameters given the measurement data, according to Bayes’ theorem. This posterior parameter distribution quantifies the updated probabilities given prior knowledge of the parameters along with the model’s agreement with the observed data (Mitra and Hlavacek, 2019; Linden et al., 2022).

When the Bayesian likelihood is constructed using an individual patient’s clinical data, the resulting posterior can represent the patient’s digital twin: a probability-weighted collection of parameter sets whose simulation outputs best represent the underlying patient data. While this is relatively straightforward in theory, for a model with no closed-form solution, numerical or sampling-based methods are required to approximate the likelihood function, and thus the posterior distribution (Wilkinson, 2007; Csilléry et al., 2010; Kimpton et al., 2025). Approaches such as approximate Bayesian computation, adaptive importance sampling, and various Monte Carlo or Markov chain Monte Carlo based methods can approximate the posterior distribution through repeated sampling from the prior distribution, simulation, and comparison to observed data (Csilléry et al., 2010; van Osta et al., 2021; Santos and Valero-Cuevas, 2004; Rannala, 2002; Schiavazzi et al., 2017; Vrugt, 2016). However, even with techniques designed to promote efficient sampling, this parameter estimation problem can still present significant challenges in balancing computational constraints with the required accuracy and precision for clinical decision-making (Kimpton et al., 2025).

Alternatively, one could create a sampled prior distribution of precomputed simulations up front, before considering the clinical data of any individual patient. This approach necessitates that all target patient phenotypes are represented somewhere within the samples of that prior, thus requiring that the prior distribution be a virtual population: a collection of parameter sets that spans the full range of phenotypic variability expected within the target patient population. With such a predetermined virtual population, the most time-consuming step of creating digital twins (running numerous simulations) is performed just once. Under this method, the overhead and complexity of running simulations does not scale with each patient, but rather only with each target patient population.

Towards this goal, we report on our method to develop a virtual population, first using the DiffeRential Evolution Adaptive Metropolis (DREAM) algorithm (Vrugt, 2016) to approximate a parameter distribution consistent with target population-level measurement data, and subsequently utilizing weight-based allometric scaling of the resulting parameter sets to more fully match target measurement variability. We apply this methodology to a mechanistic hemodynamic model with 23 relevant unknown parameters, using the published clinical data of healthy populations, in the form of 19 pressure and volume measurements across samples of at least 125 patients apiece, to develop a healthy virtual population. Finally, we demonstrate the virtual population’s potential utility by creating proof-of-concept digital twins for 17 patients within the Medical Information Mart for Intensive Care (MIMIC)-IV database (Johnson et al., 2023), whose data were not considered during population development, using the virtual population as a fixed prior distribution for patient-specific Bayesian inference through rapid posterior reweighting.

## 2 Materials and methods

Our methods are divided into three main sections, as diagrammed in **Figure 1**. First, we introduce the lumped-parameter cardiovascular model used as the basis for our virtual population development, along with the parameters to be estimated and the published population-level hemodynamic measurements used as targets. Second, we describe our methodology for virtual population development, beginning from parameter point estimates and global sensitivity analysis to determine the relevant parameter space. We then estimate a population posterior using the DREAM algorithm and the population-level hemodynamic measurements, followed by allometric scaling of the resulting parameter sets to arrive at the full virtual population. Finally, we describe a Bayesian inference technique which uses the virtual population as a fixed, precomputed prior distribution to efficiently obtain proof-of-concept patient-specific digital twins.

**Figure 1:**
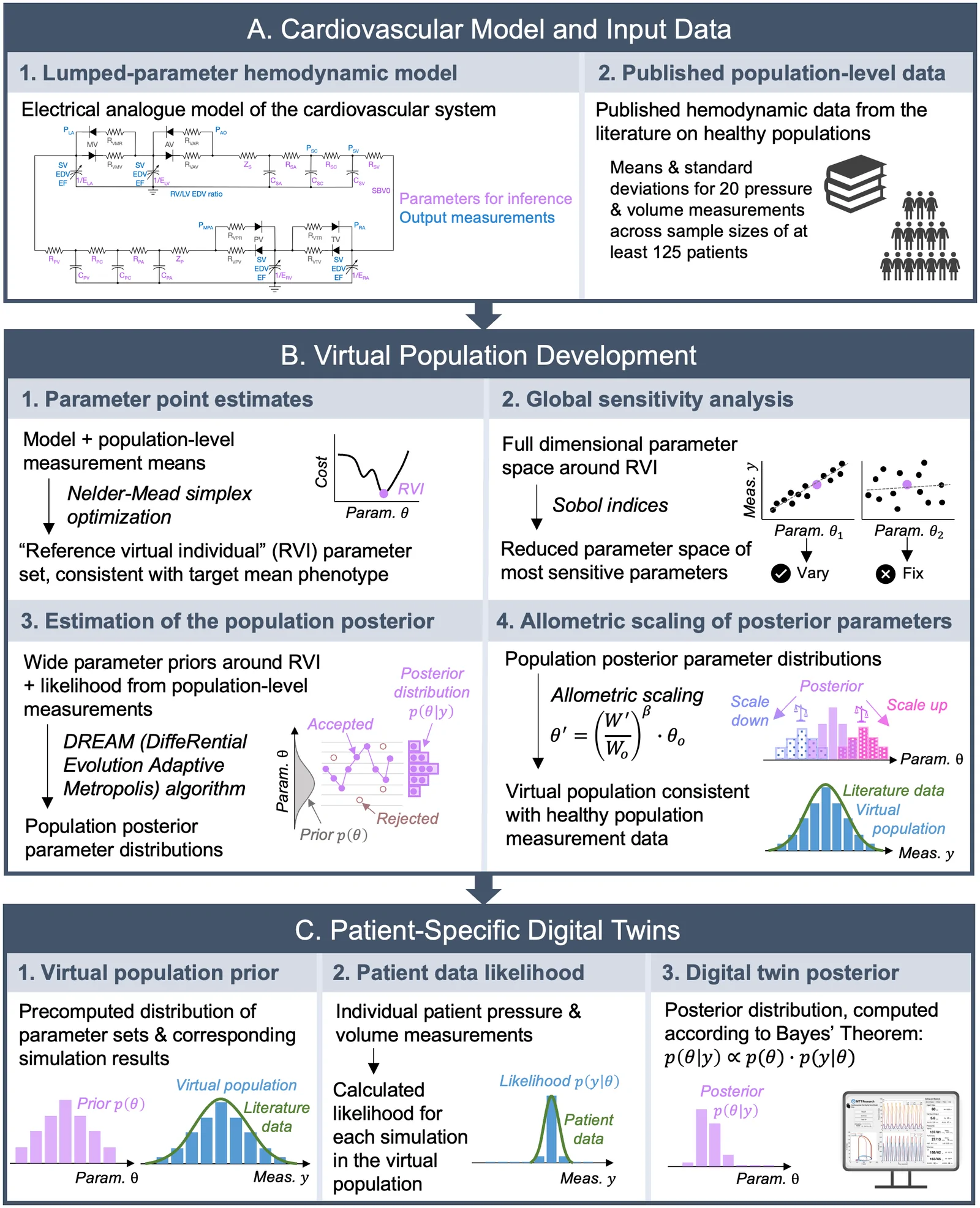
Overview of the virtual population development methodology, starting from a lumped-parameter hemodynamic model and published population-level data **(A)**. Virtual population development **(B)** utilizes parameter point estimates and a global sensitivity analysis to determine the relevant parameter space. Within this parameter space, a population posterior is approximated, followed by allometric scaling of posterior parameter sets to obtain the full virtual population. Finally, this virtual population is used as a fixed, precomputed prior distribution to perform Bayesian inference with individual patient clinical data to obtain patient-specific digital twins **(C)**.

### 2.1 Cardiovascular model and input data

#### 2.1.1 Lumped-parameter hemodynamic model

We applied our Bayesian methodologies to a mechanistic model of the cardiovascular system based on an electrical analogue representation, as diagrammed in **Figure 2**, similar to several previously published works (Doshi and Burkhoff, 2016; Shimizu et al., 2018; Matsushita et al., 2025; Santamore and Burkhoff, 1991; Burkhoff and Tyberg, 1993). This lumped-parameter model uses electrical analogies: pressures correspond to voltage, blood flow to current, and blood volume to charge. Thus, as charge stored on a capacitor generates voltage, blood volume stored within the walls of a compliant artery generates pressure. Similarly, vascular resistance is defined as pressure gradient divided by blood flow, analogous to Ohm’s law (Kokalari et al., 2013).

**Figure 2:**
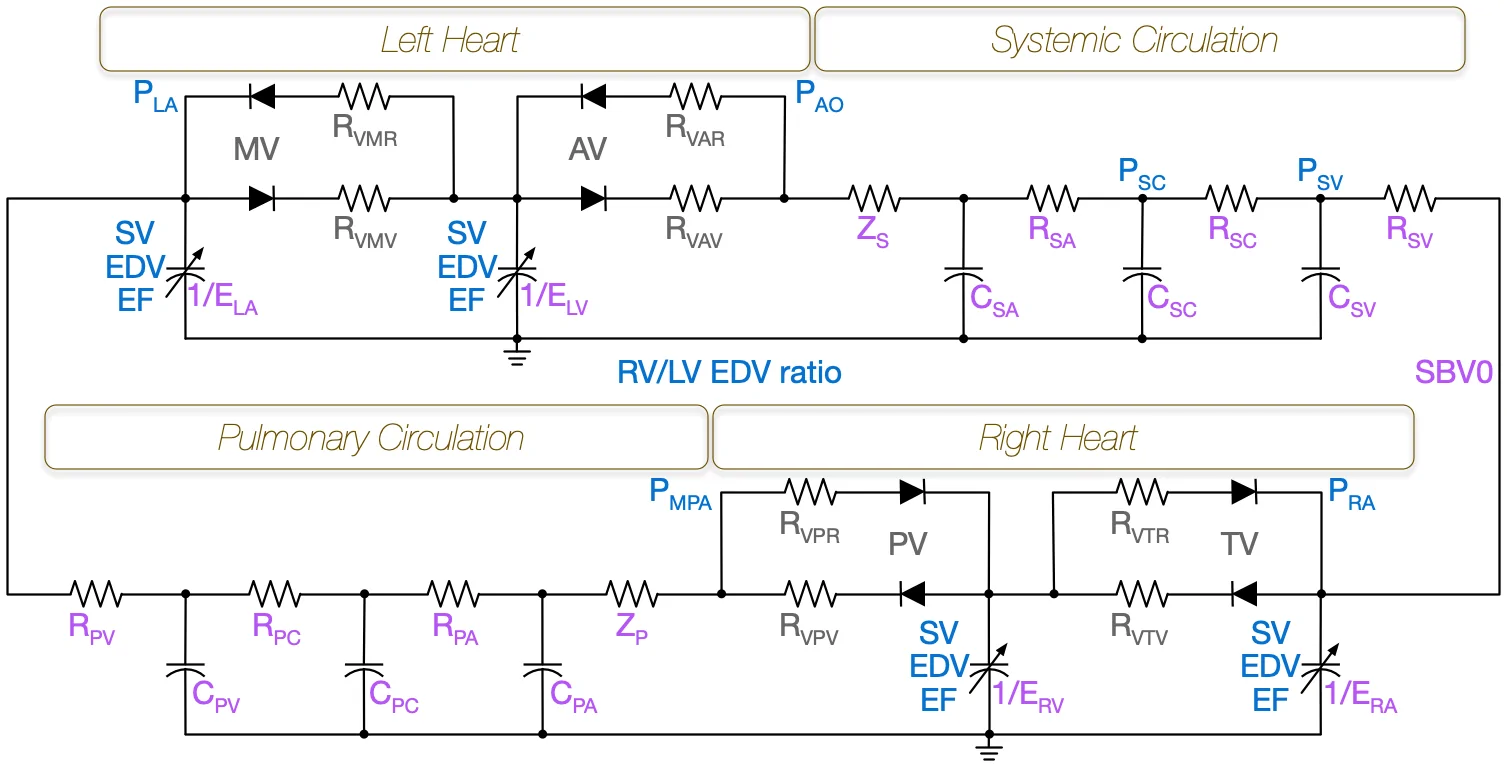
Lumped-parameter model of the cardiovascular system. Variable parameters of the model (section 2.1.1) are shown in purple, while measurements with available healthy population-level data from the literature (section 2.1.2) are shown in blue, including pressures (P) throughout the system, as well as stroke volume (SV), end-diastolic volume (EDV), and ejection fraction (EF) for each heart chamber. Resistance parameters of the four atrio-ventricular and semi-lunar valves (mitral valve MV, aortic valve AV, tricuspid valve TV, and pulmonary valve PV) were treated as constants for this investigation and are labeled in grey.

For each cardiac chamber, the contractile function that results in pressure generation is modeled as a time-varying elastance, prescribing a periodic increase in elastance during contraction (systole), followed by a decrease during relaxation (diastole) (Shimizu et al., 2018; Suga et al., 1973; Burkhoff et al., 1988; Alexander Jr et al., 2018; Stergiopulos et al., 1996). End-systolic elastances for each chamber determine the peak elastance, while the nonlinear end-diastolic pressure-volume relationship (EDPVR) governs end-diastolic volume (Burkhoff and Tyberg, 1993). Modified Windkessel vascular components are then used to represent the systemic and pulmonary vascular systems, dividing each into arterial, capillary, and venous components (Kaye et al., 2021; Burkhoff et al., 1988; Stergiopulos et al., 1996; Westerhof et al., 2009). See the **Supplementary Material** for further information on the model structure, including details of the time-varying elastance functions.

The 23 significant unknown parameters considered relevant for fitting the clinical measurement data of a healthy population were varied in this investigation, as listed in **Table 1**. Due to a lack of available measurement data within the pulmonary vasculature to disambiguate its arterial, capillary, and venous components, we chose to link the three pulmonary resistances (R_PA_, R_PC_, and R_PV_) and the three pulmonary compliances (C_PA_, C_PC_, and C_PV_), maintaining their original ratios from the initial parameter set listed in **Supplementary Table 1**. To our knowledge, comprehensive clinical data on the healthy pulmonary vasculature is lacking in the literature, in part due to the challenging and invasive techniques that would be required to obtain these measurements. Therefore, we chose to reduce the parameter space accordingly from 23 to 19 parameter distributions to be estimated.

**Table 1:** Model parameters for inference.

Parameter	Description	Units
C_PA_	Pulmonary arterial compliance	mL*/*mmHg
C_PC_	Pulmonary capillary compliance	mL*/*mmHg
C_PV_	Pulmonary venous compliance	mL*/*mmHg
C_SA_	Systemic arterial compliance	mL*/*mmHg
C_SC_	Systemic capillary compliance	mL*/*mmHg
C_SV_	Systemic venous compliance	mL*/*mmHg
LA_EDV8.5	Left atrial end-diastolic volume point (at pressure = 8.5 mmHg)	mL
LA_Ees	Left atrial end-systolic elastance	mmHg*/*mL
LV_EDV7.5	Left ventricular end-diastolic volume point (at pressure = 7.5 mmHg)	mL
LV_Ees	Left ventricular end-systolic elastance	mmHg*/*mL
RA_EDV5	Right atrial end-diastolic volume point (at pressure = 5 mmHg)	mL
RA_Ees	Right atrial end-systolic elastance	mmHg*/*mL
RV_EDV5	Right ventricular end-diastolic volume point (at pressure = 5 mmHg)	mL
RV_Ees	Right ventricular end-systolic elastance	mmHg*/*mL
R_PA_	Pulmonary arterial resistance	mmHg · s*/*mL
R_PC_	Pulmonary capillary resistance	mmHg · s*/*mL
R_PV_	Pulmonary venous resistance	mmHg · s*/*mL
R_SA_	Systemic arterial resistance	mmHg · s*/*mL
R_SC_	Systemic capillary resistance	mmHg · s*/*mL
R_SV_	Systemic venous resistance	mmHg · s*/*mL
SBV0	Initial volume distributed amongst the heart and the stressed vasculature	mL
Z_P_	Pulmonary characteristic impedance	mmHg · s*/*mL
Z_S_	Systemic characteristic impedance	mmHg · s*/*mL

#### 2.1.2 Published population-level data

Clinical measurement values from the literature on healthy adult populations were used as the measurement targets, with the data pooled across men and women where sex-specific values were available. We focused on matching per-beat measurements such as systolic and diastolic pressures, rather than matching full time-course waveforms, as these are the measurements most commonly utilized in clinical decision making (Heidenreich et al., 2022; Lang et al., 2015; Nandi et al., 2022). The published measurements fell broadly into two categories: volumes and pressures. The volume measurements included end-diastolic volume, stroke volume, and ejection fraction for each of the four cardiac chambers; additionally, the ratio of right to left ventricular (RV to LV) end-diastolic volume was used to capture the relationship between the two ventricles. Pressure measurements included systolic and diastolic aortic and pulmonary arterial (PA) pressures, and mean left and right atrial (LA and RA) pressures. Details on these 19 measurements and their literature sources are provided in **Table 2**, with target mean values and standard deviations from the literature additionally provided in **Supplementary Table 2**.

**Table 2:** Clinical measurement targets from the literature.

Measurement	Schematic identifier	Units	Reference and study population
LA end-diastolic volume LA stroke volume LA ejection fraction RA end-diastolic volume RA stroke volume RA ejection fraction	LA EDV LA SV LA EF RA EDV RA SV RA EF	mL mL % mL mL %	Petersen et al. (2017), Table 13. Healthy Caucasian adults aged 45 – 74 years from the UK Biobank, n=363 males and 432 females
LV end-diastolic volume LV stroke volume LV ejection fraction RV end-diastolic volume RV stroke volume RV ejection fraction	LV EDV LV SV LV EF RV EDV RV SV RV EF	mL mL % mL mL %	Petersen et al. (2017), Table 12. Healthy Caucasian adults aged 45 – 74 years from the UK Biobank, n=368 males and 432 females
RV to LV end-diastolic volume ratio	RV/LV EDV ratio	unitless	McCracken et al. (2023), Table 1. Healthy adults from the UK Biobank, n=15,013 males and 17,687 females
Systolic aortic pressure	P_AO_ systolic	mmHg	Wright et al. (2011), Table 3 and 4. Adults aged 18 and over with normal hypertension status from the United States, n=6,780 males and 6,313 females
Diastolic aortic pressure	P_AO_ diastolic	mmHg	Wright et al. (2011), Table 3 and 4. Adults aged 18 and over with normal hypertension status from the United States, n=6,780 males and 6,313 females
Systolic pulmonary arterial pressure Diastolic pulmonary arterial pressure Mean left atrial pressure	P_MPA_ systolic P_MPA_ diastolic P_LA_ mean	mmHg mmHg mmHg	Kovacs et al. (2009), Table 1. Healthy volunteers, n=882
Mean right atrial pressure	P_RA_ mean	mmHg	Reddy et al. (2019), Table 1. Adults presenting with non-cardiac dyspnea, n=55 males and 70 females
Mean mid-capillary pressure - *used only to estimate the reference virtual individual (RVI)*	Mean of P_SC_ and P_SV_	mmHg	Morissette (1977), Table 1. Colloid osmotic pressure for healthy adults, n=300 (used as a surrogate for mean mid-capillary pressure)

To develop a healthy human virtual population, we sought out published literature for reasonably large sample sizes of healthy adults, with no noted cardiovascular or other diseases. Our goal was to find measurements that would correspond to quantities well-distributed throughout our cardiovascular model, such that parameters of interest could reasonably be expected to be identifiable. However, invasive hemodynamic measurements are not routinely taken for healthy individuals, so where necessary, we utilized data for non-healthy patients with no noted cardiovascular disease related to the measurement under study. For example, mean RA pressure was obtained for patients with exertional dyspnea who underwent invasive hemodynamic exercise testing and were found to have no cardiac cause for their symptoms (Reddy et al., 2019).

Note that we additionally used a measurement of blood colloid osmotic pressure (Morissette, 1977) as a surrogate for mid-capillary pressure during estimation of an initial parameter point estimate consistent with a target mean phenotype (see section 2.2.1). This mid-capillary pressure surrogate was intended to ensure that the systemic vascular parameters produce simulated pressures consistent with capillary exchange. Colloid osmotic pressure can be used to approximate blood pressure midway through the capillaries, where the direction of exchange switches and therefore blood pressure and colloid osmotic pressure equalize (Darwish and Lui, 2023). However, this measurement is rarely available in clinical practice, so explicitly matching variability in this measurement was not relevant to our development of a representative healthy virtual population.

### 2.2 Virtual population development

Our virtual population development methodology consisted of four main steps, as depicted in panel B of **Figure 1**. First, we obtained parameter point estimates consistent with the target mean phenotype of the population; the resulting parameter set comprised a “reference virtual individual” (RVI), a parameter set consistent with an average healthy phenotype. Second, we performed a global sensitivity analysis around this RVI parameter set to determine the most impactful parameters to vary in the virtual population. For this subset of parameters to vary, we then estimated a population posterior consistent with the population-level measurement means and standard deviations. Finally, we utilized allometric scaling to systematically scale parameter sets to increase the virtual population variability in chamber volumes while maintaining our existing pressure distributions. Each of these steps will be explained in more detail below.

#### 2.2.1 Parameter point estimates

Before determining parameter distributions to map to target population-level measurement variability, we obtained parameter point estimates to map a single parameter set to target mean measurement values. For this analysis, the simulated heart rate was fixed to 70 bpm to correspond to the approximate mean of the healthy populations detailed in **Table 2**. Additionally, because stressed blood volume is challenging to estimate without specially-designed experimental procedures, we fixed the value of the SBV0 parameter to the initial value reported in **Supplementary Table 1**, based on measured values reported in Fudim et al. (2022). Despite being fixed for these purposes, we later vary SBV0 when obtaining the healthy population posterior and the ultimate virtual population, adding back uncertainty on this difficult to measure parameter.

For the remaining 18 parameters of interest, the Nelder-Mead simplex algorithm (Nelder and Mead, 1965) was used to simultaneously fit all parameters to minimize a cost function, defined as the sum of a normalized root mean square error (NRMSE) of the simulation outputs versus measurements targets and a penalty function to enforce parameter ranges. Specifically, NRMSE was computed across all *M* = 20 measurements as shown in Equation 2:

(2) $$\text{NRMSE}=\sqrt{\frac{1}{M}\overset{M}{\underset{i=1}{\sum }}{\left(\frac{y_{sim,i}-\mu_{i}}{\sigma_{i}}\right)}^{2}}$$

where, for measurement *i* ∈ {1*,…,M*}, *y_sim,i_* is the simulation result, and *µ_i_* and *σ_i_* are the mean value and the standard deviation of the population data from **Supplementary Table 2**.

To manage the high dimension of a parameter space, an iterative fitting approach was used in which parameter ranges were repeatedly reduced to obtain higher precision estimates, with multiple parameter fits performed at each round, each starting from different randomly selected values within the ranges. For details of this procedure, and the formulation of the penalty function to enforce parameter ranges, please see the **Supplementary Material**. Upon convergence, when the top 10 of 50 repeated parameter fits all fell within less than 1% of their corresponding initial range, the mean of those top 10 final parameter fits was taken as the RVI parameter set.

#### 2.2.2 Global sensitivity analysis

Global sensitivity analysis was performed around the RVI parameter set to determine the collection of parameters to be varied in the virtual population. This step was critical to balance the tradeoff between exploration of all axes of parameter uncertainty versus realistic computational and sampling density constraints. We utilized Sobol’s method to decompose model output variance into the portion dependent on each parameter (Sobol, 1993; Zhang et al., 2015).

We computed the total Sobol indices for each of the *N* = 19 independent parameters defined in **Table 1** relative to the 19 measurements defined in **Table 2**, testing each parameter in a range of ±50% around their RVI value. Based on guidance in the literature, we chose a sample size of *n* = 10,000 simulations per parameter, for a total simulation cost of (*N* + 2) · *n* = 210,000 simulations (Saltelli, 2002; Marelli et al., 2026; Zhang et al., 2015). From the global sensitivity results, we chose to vary in the virtual population all parameters with a total Sobol index greater than 0.05 for at least one measurement (Zhang et al., 2015); in other words, we considered any parameter responsible for at least 5% of the overall variability in at least one measurement to be significant to the virtual population.

#### 2.2.3 Estimation of the population posterior

Across the multi-dimensional parameter space determined by the global sensitivity analysis, we approximated a posterior distribution consistent with the target population-level measurements using the DiffeRential Evolution Adaptive Metropolis (DREAM) algorithm, specifically the DREAM_(ZS)_ variant (Vrugt et al., 2009; Laloy and Vrugt, 2012). The DREAM algorithm leverages multi-chain methods to improve the efficiency and convergence speed relative to traditional Markov chain Monte Carlo (MCMC) methods (Vrugt et al., 2009; Vrugt, 2016), utilizing both differential evolution (Storn and Price, 1997) and self-adaptive randomized subspace sampling to improve performance, particularly in high-dimensional, nonlinear, or multimodal problems (Vrugt et al., 2009). By incorporating sampling from an archive of past states, the DREAM_(ZS)_ variant further improves convergence speed even with a smaller number of chains (Laloy and Vrugt, 2012).

The parameter samples generated through the DREAM algorithm ultimately converge to the target posterior distribution p(θ|y), where *θ* represents model parameters and *y* represents measurements corresponding to model outputs. The posterior is defined by Bayes’ theorem in Equation 3:

(3) p(θ|y)∝p(θ)·p(y|θ)

The algorithm requires a prior distribution p(θ) and a function to calculate the likelihood p(y|θ) of each parameter proposal *θ*, where sampling and simulation are used to compute the likelihood for discrete parameter samples (Vrugt, 2016).

We formulated the prior as a broad distribution around the RVI parameter set obtained in section 2.2.1, such that the prior for each parameter was a normal distribution with both mean and standard deviation equal to the corresponding RVI parameter value. By setting both the mean and standard deviation to the same value, we encouraged the posterior distribution to center near this RVI parameter set while still allowing significant exploration in other areas of the parameter space; see the **Supplementary Material (section 2.3.2)** for further analysis regarding this choice of prior. Additionally, parameter range cutoffs were enforced according to the ranges in **Supplementary Table 1**, truncating the Gaussian prior distributions.

As no general analytic solution exists for the likelihood in the case of our model, the likelihood at a particular parameter set must instead be calculated by first running the simulation to determine the resulting model outputs. Then, from those outputs, the likelihood can be computed as the joint probability of the simulation outputs according to independent normal distributions with each literature mean and standard deviation for the 19 measurements of interest, as defined in **Supplementary Table 2**.

The DREAM algorithm was run with six chains for 20,000 generations, at which point convergence was assessed based on the Gelman-Rubin (GR) diagnostic (Gelman and Rubin, 1992; Vrugt, 2016). As they satisfied the convergence criteria, parameter samples from the final 10,000 generations, for all six chains, were kept as the approximation of the posterior distribution, resulting in 60,000 parameter samples within the posterior.

#### 2.2.4 Allometric scaling of posterior parameters

The posterior distribution as obtained in the previous section is, by definition, consistent with the supplied prior and likelihood. However, this does not guarantee that the posterior’s corresponding distribution of simulated outputs fully matches the variability of the target patient population. First, by providing non-uniform priors informed by the RVI parameter set, we slightly decrease posterior variability beyond the constraints enforced by the likelihood. Second, by having multiple measurement targets constraining the same aspects of the system (e.g., end-diastolic volume, stroke volume, and ejection fraction for each cardiac chamber), these measurement distributions are likely to be overconstrained or narrowed in the posterior relative to the literature targets. We explicitly chose to include all measurements because matching the variability in each is important to our virtual population, as will be discussed further in section 4.1.1 and in the **Supplementary Material** (section 2.3.1), but it necessitates special handling for chamber volume variability.

As a result, we included a final step in virtual population development to utilize allometric scaling principles to increase the variability on the volume measurements while leaving pressure distributions relatively unchanged. Allometric scaling describes the ways that physiological quantities scale, often nonlinearly, with body size (Equation 4):

(4) $$Y=\alpha \cdot M^{\beta }$$

where *Y* is the quantity being scaled, *α* is the allometric coefficient, *M* is body mass, and *β* is the allometric exponent. Importantly, the standard allometric exponent *β* is approximately 1 for cardiac volumes, indicating linear scaling with body weight, and approximately 0 for pressures, indicating no scaling with body weight (Günther, 1975; Günther and Morgado, 1996; West et al., 1997).

However, rather than scaling model outputs, we utilized allometric scaling of model parameters Equation 5 to scale each parameter sample making up the population posterior of section 2.2.3:

(5) $$\theta^{\prime }={\left(\frac{W^{\prime }}{W_{0}}\right)}^{\beta }\cdot \theta_{0}$$

where *θ*^′^ is the scaled parameter value resulting from the original value *θ*_0_, scaled based on new weight *W*^′^ relative to original weight *W*_0_. Based on the literature reference for our chamber volume measurements, we set *W*_0_ = 70 kg (corresponding to the approximate population mean), and selected *W*^′^ = 50..(5)..90 kg: samples every 5 kg within a range of approximately -2 to 2 standard deviations around the mean (Petersen et al., 2017). Allometric exponents *β* for our parameters were defined as follows (Günther, 1975; West et al., 1997):

- End-systolic elastance (Ees) parameters: *β* = −1
- Volume (EDV and SBV0) and compliance (C) parameters: *β* = 1
- Resistance (R) and characteristic impedance (Z) parameters: *β* = −0.75

While heart rate also scales allometrically with weight (Günther, 1975) we instead opted to handle heart rate separately at this stage since it is directly measured and reported in the population-level literature, and it can vary significantly within and across individuals, independent of body size (Quer et al., 2020). As with our choice of weight for allometric scaling, we chose to simulate each posterior parameter set with heart rates of 50..(5)..90 bpm, corresponding to every 5 bpm within a range of approximately -2 to 2 standard deviations around the reported mean from Petersen et al. (2017).

Combining the nine discrete weight scalings and nine heart rates, each posterior parameter set from section 2.2.3 was simulated a total of 81 times to cover all combinations. The resulting 4.86 million parameter sets were then combined using a probability-weighted approach, based on the Gaussian distributions of body weight and heart rate with the approximate reported population-level mean and standard deviation: 70 ± 10 kg and 70 ± 10 bpm (Petersen et al., 2017).

This final virtual population was evaluated quantitatively for coverage of the target clinical population based on percentile ranges of the resulting simulation output distributions and the literature targets. Specifically, we computed the percentage of the target distribution which was covered by the 5th to 95th percentile of the simulated measurements. The 5th to 95th percentile represents 90% of a distribution, so we computed the percent of expected coverage as the resulting fractional coverage value divided by 0.9. For this analysis, good coverage is considered to be achieved if all measurements fall between 80% and 110% of target coverage, with the upper bound reflecting that a measurement markedly beyond 100% coverage is not desirable in this context; such a value would indicate that coverage of other measurements is only occurring in conjunction with values outside of the physiological range for any measurement substantially exceeding 100% coverage.

### 2.3 Patient-specific digital twins

Finally, to demonstrate a proof-of-concept application of the virtual population, we developed 17 patient-specific digital twins using Bayesian inference with the virtual population as a fixed, precomputed prior distribution. Note that the present work does not directly incorporate certain key aspects of a true digital twin, namely dynamic updating and prediction of treatment responses (National Academies of Sciences, Engineering, and Medicine, 2024). However, as will be further covered in the Discussion (section 4.3), these features are compatible with the framework presented here.

#### 2.3.1 Virtual population prior

In contrast to our Bayesian approximation approach in section 2.2.3 for population-level inference, for patient-specific Bayesian inference we utilized the existing, precomputed virtual population of section 2.2.4 as a fixed prior distribution, *p_vp_*(*θ*); thus, the scaled posterior representing the population becomes the prior. Concretely, the prior distribution consisted of the collection of parameter sets Θ*_vp_* making up the virtual population, with probabilities assigned according to body weight and heart rate distributions in the target patient population, as described previously.

#### 2.3.2 Patient data likelihood

The patient data likelihood was constructed using available clinical measurements corresponding to a subset of the published measurement data available for the healthy population. Due to the discrete, sampled nature of the prior distribution, the likelihood was calculated for only those sampled parameter sets: $p(y_{patient}|\theta ){|}_{\theta \in {\text{Θ}}_{vp}}$. Specifically, the joint probability of all simulation outputs was calculated according to independent normal distributions centered at patient-specific clinical measurement values, with a standard deviation set to one-quarter of the corresponding population-level standard deviation. This value was used as a proxy for measurement uncertainty, including both intra-individual variability and measurement error.

Detailed hemodynamic assessment is not often performed for truly healthy individuals, but for the context of our hemodynamic model, we selected patients from the Medical Information Mart for Intensive Care (MIMIC)-IV database (Johnson et al., 2023, 2024; Goldberger et al., 2000) who were in the intensive care unit for non-cardiovascular reasons and who had hemodynamic measurements within healthy ranges. Specifically, we selected patients hospitalized after liver transplant, for whom the following measurements were simultaneously available within the same one-hour period: systolic and diastolic arterial pressure, systolic and diastolic pulmonary artery pressure, central venous pressure (a surrogate for mean RA pressure), cardiac output, and heart rate. Seventeen unique patients satisfied the selection criteria; see **Supplementary Section 1.4** for further details of these criteria.

To maintain consistency with the population-level measurements, we calculated stroke volume from cardiac output and heart rate and compared this value against simulated LV stroke volume rather than directly using cardiac output in the likelihood. Note that while patient weight was available for most patients, it was not used for inference. Instead, the weight parameter used for allometric scaling remained a latent grid variable as it was intended as a population-level mathematical tool, rather than a physiological value that would directly correspond to an individual patient’s observed weight. Finally, neither these specific patients nor the MIMIC-IV database more broadly were considered during the development of the virtual population, allowing these data to serve as an independent cohort against which to test the utility of our virtual population.

#### 2.3.3 Digital twin posterior

Posterior distributions for each of the 17 patients were calculated in Equation 6 based on Bayes’ theorem and the previously described virtual population prior and patient data likelihood:

(6) $$p(\theta |y_{patient})\propto p_{vp}(\theta )\cdot p(y_{patient}|\theta ){|}_{\theta \in {\text{Θ}}_{vp}}$$

This posterior, a weighted collection of parameter sets and corresponding simulation results, comprised the patient’s digital twin.

Finally, goodness of fit of each posterior distribution to the target patient-specific clinical data was assessed (Equation 7) with a mean absolute percentage error (MAPE) of the posterior measurement means:

(7) $$\text{MAPE}=\frac{100}{M}\sum_{i=1}^{M}\left|\frac{{\bar{y}}_{twin,i}-y_{patient,i}}{y_{patient,i}}\right|$$

where ${\bar{y}}_{twin,i}$ is the weighted mean of measurement *i* in the digital twin posterior distribution and $y_{patient,i}$ is the patient’s corresponding clinical measurement target for the *M* = 6 patient-specific measurements defined above. To additionally evaluate the probabilistic aspect of the posterior distributions, the percentage of measurements where the target value lies within the equal-tailed 90% credible interval (5th to 95th percentile) of the corresponding posterior distribution was computed.

This analysis is intended to demonstrate feasibility of using the precomputed virtual population as a fixed prior for patient-specific inference; as such, the goodness of fit metrics evaluate the ability of a posterior selected from within the virtual population prior to match individual patient data that was not considered during development of the virtual population. Importantly, this does not constitute a validation of the digital twins themselves, in terms of predictions of withheld measurements, later time points, or intervention responses. However, it does demonstrate the feasibility of rapid posterior reweighting from within a precomputed virtual population prior.

### 2.4 Code implementation

Downstream analysis of the population posterior and MIMIC-IV data handling were performed in R (R Core Team, 2025) using the posterior software package (Bürkner et al., 2026) and the RICU software package (Bennett et al., 2023), respectively. The lumped-parameter hemodynamic model was implemented using MATLAB and Simulink computing software (The MathWorks Inc., 2024), then compiled (with a fixed step size of 2 ms) for improved performance. All other analyses were performed in MATLAB. Global sensitivity analysis utilized the UQLab software package (Marelli and Sudret, 2014; Marelli et al., 2026), while estimation of the healthy population posterior utilized the DREAM software package (Vrugt et al., 2009; Vrugt, 2016). All analysis and computational speed benchmarking were performed on an Apple M3 MacBook Pro (12 cores, 36 GB unified memory, 512 GB storage).

### 2.5 Ethical considerations

The MIMIC-IV database used to obtain patient-specific clinical data is a de-identified, publicly available dataset, and its use for research has been approved by the Institutional Review Board (IRB) of the Massachusetts Institute of Technology (MIT) and Beth Israel Deaconess Medical Center (BIDMC). As the data are anonymized, the BIDMC IRB has waived the requirement for informed consent (Johnson et al., 2023).

Additionally, all population-level data were taken directly from the primary text of previously published manuscripts. These literature data were in the form of summary metrics (measurement means and standard deviations) across the participants of each original study. As these data were already publicly available, no ethical approval was required for our usage of the data.

## 3 Results

### 3.1 Virtual population development

#### 3.1.1 Parameter point estimates

The iterative parameter fitting process to determine a single RVI parameter set required 12 rounds to satisfy the convergence criteria, as depicted in **Supplementary Figure 1**. With the mean parameter set across the best 10 of 50 fits at this final round (see **Table 3** for parameter values and **Table 4** for corresponding simulation results), the NRMSE of simulation results relative to the healthy population mean measurement targets was 0.0126, calculated according to Equation 2. In other words, simulation results differed from measurement targets by an average of roughly 1.3% of the corresponding population-level standard deviations, representing an accurate simultaneous fit to all target mean measurement values.

**Table 3:** Final parameter point estimates, or the RVI parameter set, rounded to three significant figures.

Parameter	Fitted value	Units
${\text{C}}_{\text{PA}}$	3.83	mL/mmHg
${\text{C}}_{\text{PC}}$	3.96	mL/mmHg
${\text{C}}_{\text{PV}}$	4.11	mL/mmHg
${\text{C}}_{\text{SA}}$	1.44	mL/mmHg
${\text{C}}_{\text{SC}}$	9.53	mL/mmHg
${\text{C}}_{\text{SV}}$	100	mL/mmHg
LA_EDV8.5	61.7	mL
LA_Ees	0.379	mmHg/mL
LV_EDV7.5	135	mL
LV_Ees	1.96	mmHg/mL
RA_EDV5	71.2	mL
RA_Ees	0.110	mmHg/mL
RV_EDV5	152	mL
RV_Ees	0.284	mmHg/mL
${\text{R}}_{\text{PA}}$	0.00716	mmHg·s/mL
${\text{R}}_{\text{PC}}$	0.0179	mmHg·s/mL
${\text{R}}_{\text{PV}}$	0.00990	mmHg·s/mL
${\text{R}}_{\text{SA}}$	0.572	mmHg·s/mL
${\text{R}}_{\text{SC}}$	0.215	mmHg·s/mL
${\text{R}}_{\text{SV}}$	0.0473	mmHg·s/mL
SBV0	1860	mL
${\text{Z}}_{\text{P}}$	0.0226	mmHg·s/mL
${\text{Z}}_{\text{S}}$	0.0373	mmHg·s/mL

Note that the pulmonary capillary and venous parameters (R_PC_, R_PV_, C_PC_, C_PV_) were linked to their corresponding arterial parameters (R_PA_ and C_PA_, respectively) as described in section 2.1.1, and SBV0 was fixed rather than fitted, as described in section 2.2.1.

**Table 4:** Simulation results versus literature measurement targets for the RVI parameter set, with all simulation results rounded to three significant figures.

Measurement	Literature target	Simulated	Units
LA end-diastolic volume	66	66.3	mL
LA stroke volume	40	39.8	mL
LA ejection fraction	60	60.0	%
RA end-diastolic volume	80	79.8	mL
RA stroke volume	35	35.1	mL
RA ejection fraction	44	44.0	%
LV end-diastolic volume	143	143	mL
LV stroke volume	85	85.5	mL
LV ejection fraction	60	59.9	%
RV end-diastolic volume	154	153	mL
RV stroke volume	85	85.4	mL
RV ejection fraction	56	55.9	%
RV/LV end-diastolic volume ratio	1.07	1.07	unitless
Systolic aortic pressure	117	117	mmHg
Diastolic aortic pressure	69	69.0	mmHg
Systolic PA pressure	20.8	20.8	mmHg
Diastolic PA pressure	8.8	8.80	mmHg
Mean LA pressure	8.0	8.00	mmHg
Mean RA pressure	5	5.00	mmHg
Mean mid-capillary pressure	20.5	20.5	mmHg

#### 3.1.2 Global sensitivity analysis

Global sensitivity analysis around the RVI parameter set found that 13 parameters had a total Sobol index above the sensitivity threshold of 0.05 for at least one measurement: C_SA_, C_SV_, LA_Ees, LA_EDV8.5, LV_Ees, LV_EDV7.5, RA_Ees, RA_EDV5, RV_Ees, RV_EDV5, R_SA_, R_SV_, and SBV0. The remaining six parameters (C_PA_, C_SC_, R_PA_, R_SC_, Z_P_, and Z_S_) had no Sobol indices above the threshold, and therefore were fixed at their RVI parameter value for the remainder of the virtual population development process. See **Supplementary Figure 2** for further details of the Sobol indices.

#### 3.1.3 Population posterior

The healthy population posterior approximated with the DREAM algorithm revealed posterior parameter distributions that peak at or near the RVI parameter set, but with narrower distribution widths than would be produced just on the basis of the imposed prior knowledge, as depicted in **Figure 3** in grey (“Population Posterior”). This lends support that the prior formulation functioned as intended: it encouraged the posterior parameter distributions to center around a parameter set consistent with target population-level mean measurement values, without overly constraining parameter variability. See the **Supplementary Material** for further analysis of the sensitivity of posterior results to this choice, along with relevant Bayesian diagnostics for the population posterior.

**Figure 3:**
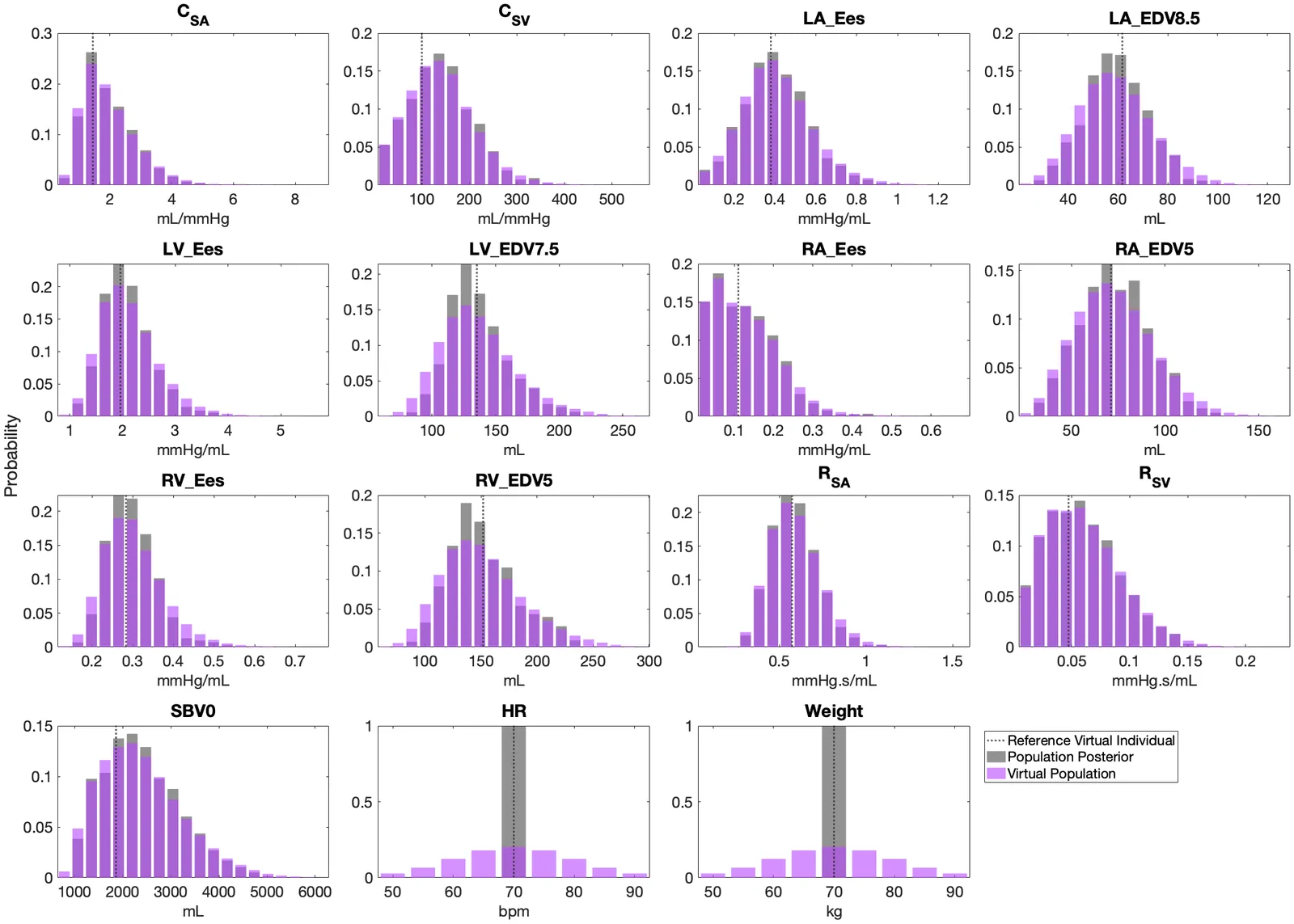
One-dimensional parameter marginal distributions for the preliminary population posterior (grey) and the final allometrically-scaled virtual population (purple). The dashed vertical line in each plot highlights the reference virtual individual (RVI) parameter value. Parameters include the 13 unknown parameters with distributions estimated in the preliminary population posterior, along with the two parameters systematically varied to expand the population posterior into the final virtual population: heart rate (HR) and body weight.

Nevertheless, the resulting measurement distributions, shown in grey in **Figure 4**, underrepresent variability in several measurements, most notably ventricular end-diastolic and stroke volumes.

**Figure 4:**
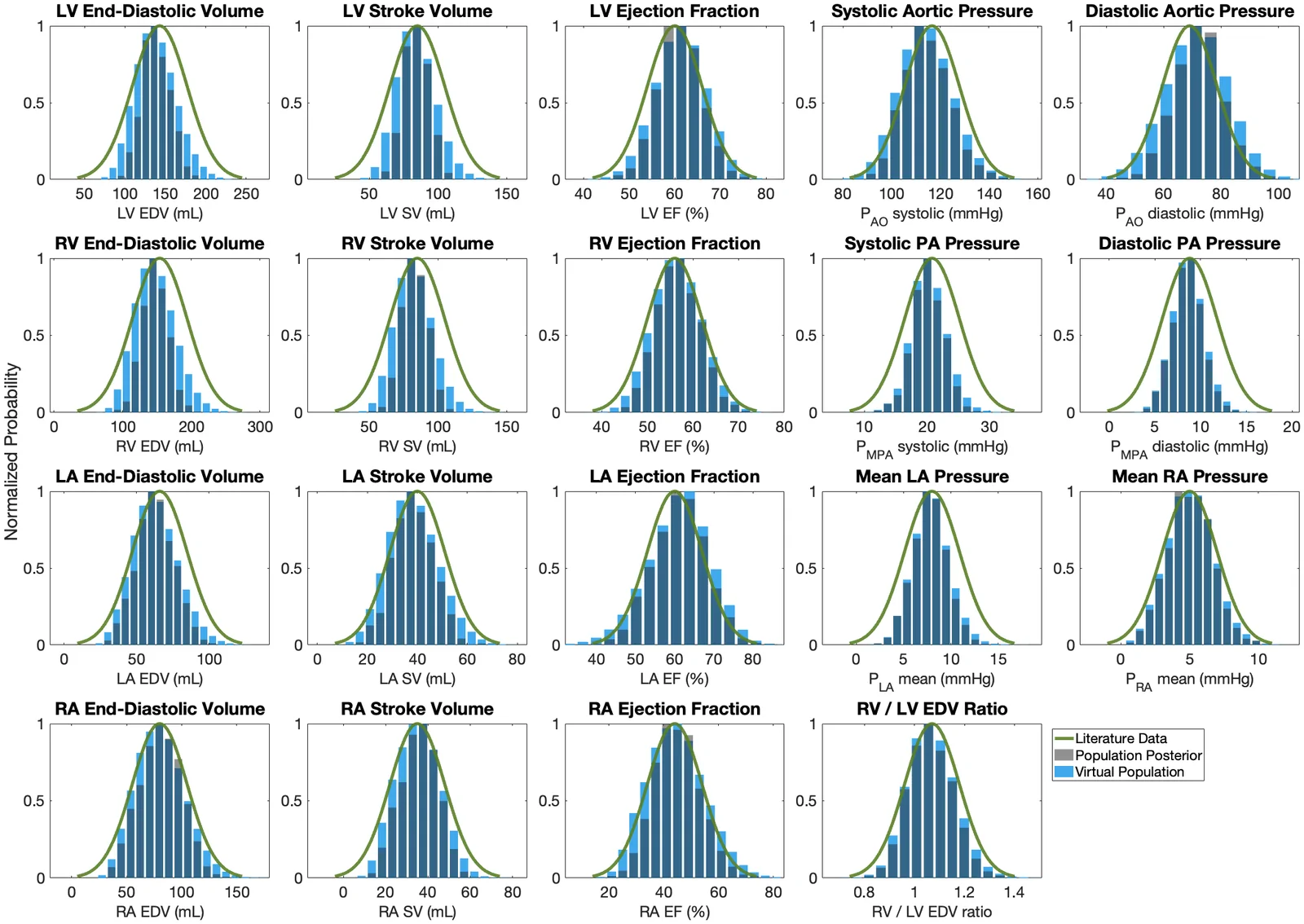
Model output measurement distributions corresponding to the preliminary population posterior (grey) and the final allometrically-scaled virtual population (blue). Note that the preliminary population posterior is generally narrower than the full virtual population, and thus is not typically visible by itself in grey; instead, that distribution is most evident in the navy blue color formed by the two distributions’ overlap. Green Gaussian curves highlight the population-level measurement target data from the literature. For ease of comparison, all histograms and Gaussian curves are normalized to have a consistent peak amplitude of 1.

#### 3.1.4 Allometrically-scaled virtual population

To improve matching of the target population-level measurement distributions and thus create a more comprehensive virtual population, we then allometrically scaled each of the posterior parameter sets from the previous section and simulated them at a range of heart rates, as described in section 2.2.4. The resulting change in parameter marginal distributions is relatively subtle for most parameters, as depicted in purple (“Virtual Population”) in **Figure 3**. Most notably, the distributions for heart rate (HR) and weight change from a single value to Gaussian distributions over the tested range of values, while other parameter distributions show a slight widening.

Despite the appearance of only relatively minor changes to the one-dimensional posterior parameter marginal distributions, the incorporation of allometric scaling and a range of heart rates visibly improved matching of the target population-level volume variability, as shown in blue in **Figure 4**. Here we can see that most pressure distributions are relatively unchanged by allometric scaling, as is predicted by allometric theory, which typically finds little to no scaling of pressures with body weight (Günther, 1975).

Quantitative comparison of the observed distributions of simulation outputs against the literature targets using the percentile-based metric defined in section 2.2.4 finds that our percentage of expected coverage ranges from 74.6% to 104.3%, as detailed in **Table 5**. Two measurements achieve less than 80% of the expected coverage: systolic and diastolic pulmonary arterial pressures. Six measurements achieve 80 – 90% of the expected coverage: mean LA pressure, LV and RV end-diastolic and stroke volumes, and RA stroke volume. The remaining 11 measurements were well represented in the virtual population, capturing 90 – 105% of the expected coverage.

**Table 5:** Virtual population 5th to 95th percentile ranges and percent of expected coverage relative to the target distribution.

Measurement	Virtual population 5th – 95th percentile range	% of expected coverage
LA end-diastolic volume	39.1 – 90.3	91.2%
LA stroke volume	22.7 – 55.7	96.1%
LA ejection fraction	47.2 – 73.4	104.3%
RA end-diastolic volume	46.4 – 119	94.5%
RA stroke volume	18.6 – 52.6	89.8%
RA ejection fraction	28.2 – 62.7	101.4%
LV end-diastolic volume	99.9 – 182	85.7%
LV stroke volume	62.5 – 108	83.2%
LV ejection fraction	52.1 – 70.2	95.9%
RV end-diastolic volume	103 – 199	85.0%
RV stroke volume	61.5 – 106	81.7%
RV ejection fraction	47.9 – 65.5	94.9%
RV/LV end-diastolic volume ratio	0.92 – 1.23	93.5%
Systolic aortic pressure	96.7 – 132	97.7%
Diastolic aortic pressure	53.3 – 89.0	103.5%
Systolic PA pressure	15.9 – 25.1	77.5%
Diastolic PA pressure	5.75 – 11.6	74.6%
Mean LA pressure	4.78 – 11.3	81.8%
Mean RA pressure	2.27 – 7.87	93.1%

All ranges are displayed here to three significant figures, and all percent coverage values are rounded to one decimal place.

### 3.2 Patient-specific digital twins

Finally, patient-specific digital twins were obtained as Bayesian posterior distributions calculated from the precomputed virtual population prior. For the 17 selected MIMIC-IV patients with non-cardiovascular cause for hospitalization, posterior distributions on the parameters were successfully obtained with generally good accuracy, visually matching the target patient-specific clinical measurements, as shown in **Figure 5**. The mean absolute percentage error across measurements (Equation 7) for the patients ranges from 0.71% to 8.61%, with a mean across patients of 2.89%; please see **Supplementary Table 5** for full reporting of the MAPE of each digital twin. Additionally, 96% of the target patient measurements fell within the equal-tailed 90% credible interval of the corresponding posterior measurement distribution, meaning that the plotted credible intervals of **Figure 5** cross the dashed identity line.

**Figure 5:**
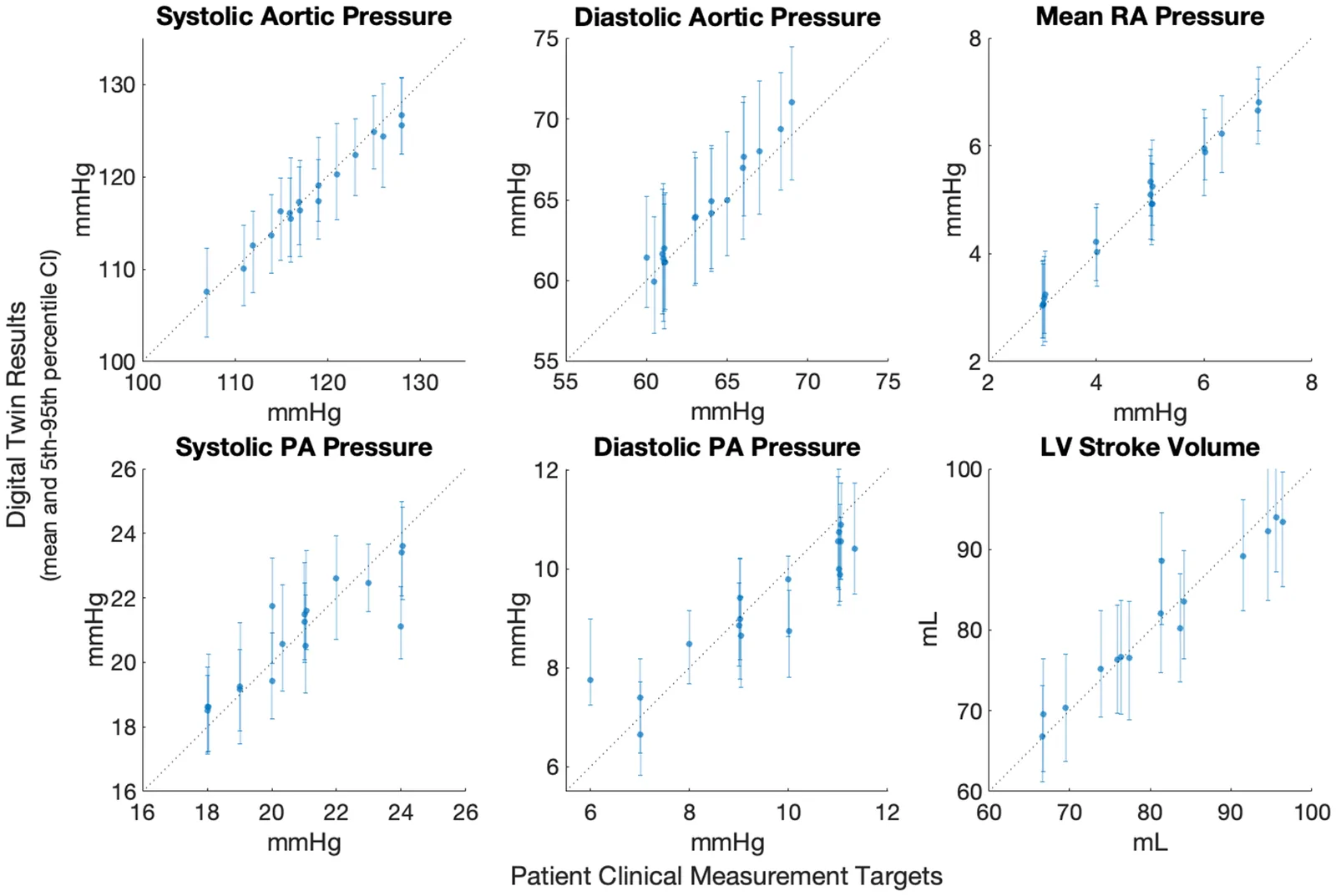
Scatter plot of patient clinical measurement targets versus digital twin results (mean and 5th-95th percentile credible interval CI), with a dashed identity line in black for reference. Note that some patients had identical measurement values; in those cases, the plotted values on the x-axis have been shifted or “jittered” very slightly, by 0.2% of the plotted axis range, to allow better visualization of the overlapping points.

It is additionally illustrative to look at full posterior parameter and measurement distributions for a representative sample of patients, where we include for completeness the patient with the largest MAPE. In the parameter distributions of **Figure 6**, comparing the patient-specific posterior distributions (purple) to the virtual population prior (grey) demonstrates that most parameter distributions are meaningfully constrained by the applied clinical measurement data, resulting in different posterior parameter distributions across the four patients. The corresponding measurement distributions of **Figure 7** show that, where patient-specific clinical measurements were available (green Gaussian curves), the digital twin measurement distributions are generally well constrained to match those targets. The notable exceptions amongst these four patients are the systolic and diastolic pulmonary arterial pressures for patient 4 (the patient with the largest overall error). As will be discussed further in section 4.2, this patient’s results demonstrate a shortcoming in the virtual population’s ability to adequately represent the full healthy range of pulmonary pressures, in terms of both marginal and joint distributions. However, these results are relatively unsurprising given the reported virtual population percent coverage of **Table 5**, and the results as a whole demonstrate the feasibility of determining distinct digital twins from the same virtual population prior distribution. Please see the **Supplementary Material** for further characterization of the patient-specific posterior distributions.

**Figure 6:**
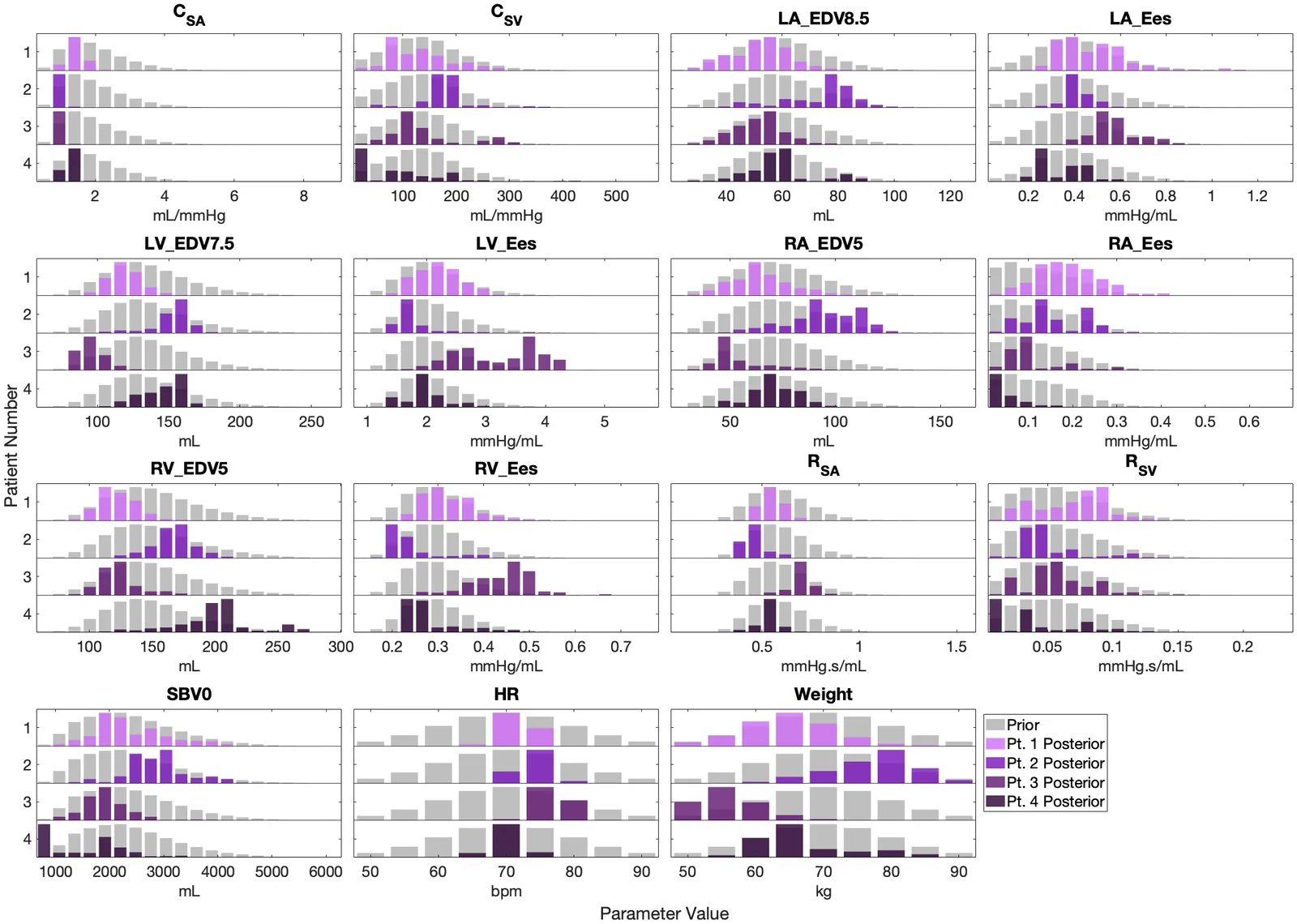
Posterior parameter marginal distributions for a sample of four patient digital twins (shades of purple), with the virtual population prior distribution (identical for all patients) plotted in grey for reference. For ease of comparison, all histograms are normalized to have a consistent peak amplitude.

**Figure 7:**
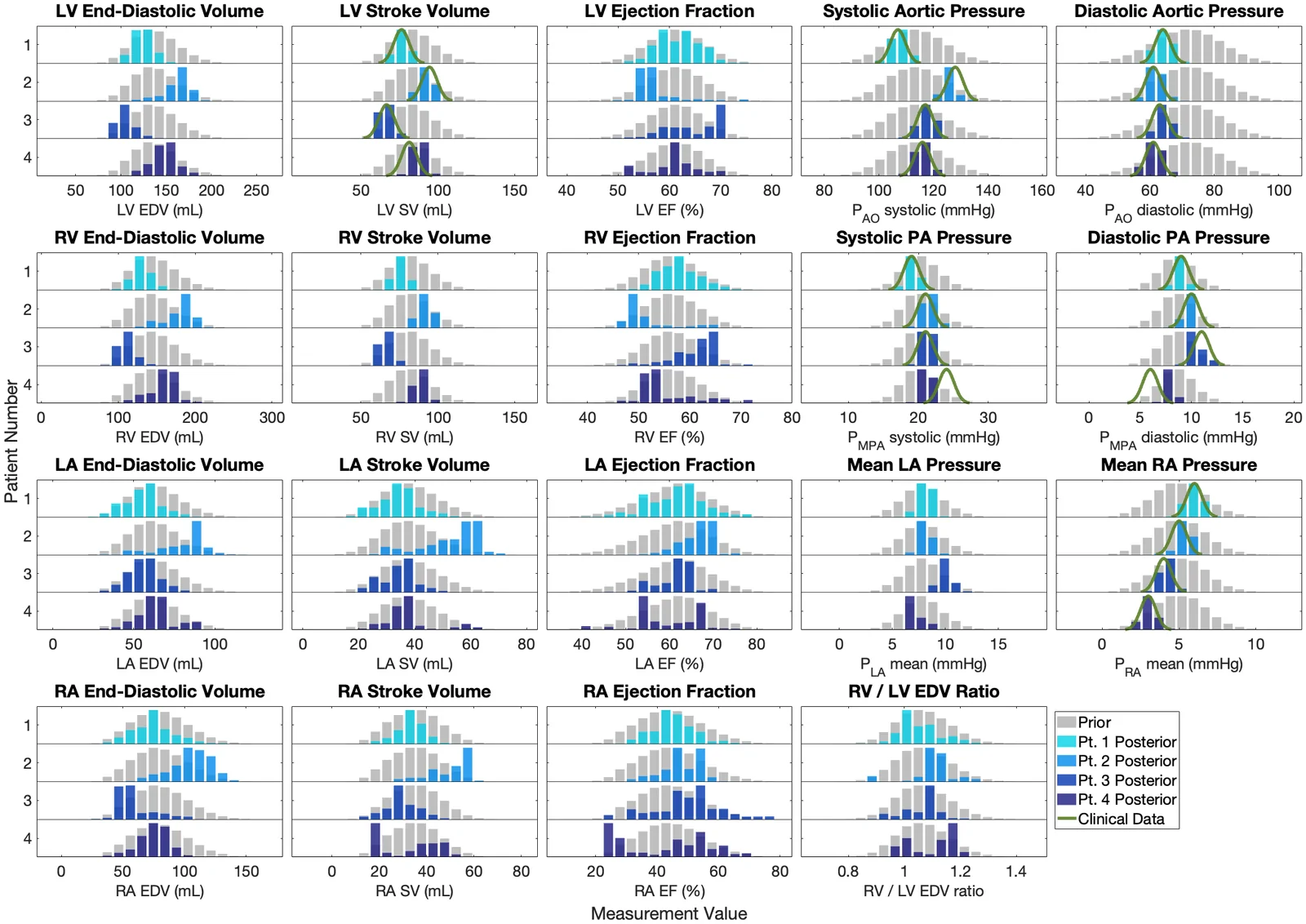
Model output measurement distributions corresponding to the sample of four patient digital twin posteriors (shades of blue), with the virtual population prior distributions (identical for all patients) plotted in grey for reference. For measurements with available patient-specific clinical data, the Gaussian curve depicts the measured value and uncertainty applied in the likelihood function. For ease of comparison, all histograms and Gaussian curves are normalized to have a consistent peak amplitude.

## 4 Discussion

This study introduces a methodology to develop a virtual population that serves as a precomputed prior distribution for patient-specific Bayesian digital twins. This virtual population is a collection of parameter sets that, when simulated through its corresponding model, spans the full range of expected phenotypic variability in a patient population of interest. The proposed methodology first obtained parameter point estimates (a reference virtual individual or RVI) consistent with target population-level means, followed by global sensitivity analysis around this RVI parameter set to determine the parameters to vary in the virtual population. It then utilized the DiffeRential Evolution Adaptive Metropolis (DREAM) algorithm to approximate the posterior distribution consistent with a likelihood constructed from population-level measurement means and standard deviations. Finally, it applied weight-based allometric scaling of the posterior parameter sets, along with varying the simulated heart rate, to expand the population posterior into a full virtual population, capable of simultaneously reproducing a sufficient portion of the variability on 19 clinical measurements of interest within a healthy patient population. The study then demonstrated a proof-of-concept use of this virtual population as the prior distribution for patient-specific Bayesian inference to obtain digital twins of 17 patients from the MIMIC-IV database.

Most established methods for virtual population or cohort generation start from a relatively small population of individual patients to generate corresponding virtual patients with a one-to-one mapping (Niederer et al., 2020; Romero et al., 2021). Subsequent random sampling from within distributions informed by those “patient-derived virtual patients” can then create an expanded cohort of “synthetic virtual patients” (Niederer et al., 2020). The virtual individuals that make up our virtual population are analogous to these synthetic virtual patients, but they are derived from Bayesian inference conditioned on population-level measurement distributions from the literature, a possibility discussed in Colebank et al. (2024), rather than first being informed by patient-derived virtual patients. Our approach to patient-specific inference then reuses this discrete virtual population as an informative prior for patient-specific inference, setting it apart from established Bayesian personalization methods and working towards a vision presented by Kimpton et al. to incorporate both patient-specific and population-level data in a “dual mechanistic-statistical framework” (Kimpton et al., 2025). This approach also bridges the gap noted by Karakasis et al. between “functionally similar digital twins [ … ] constrained by cohort-specific observations” and “functionally equivalent digital twins” personalized to an individual patient (Karakasis et al., 2025a), providing a mechanistic grounding for population-level data-driven approaches that have been utilized for diagnosis, risk stratification, and treatment improvements in conditions such as atrial fibrillation, heart failure, and hypertension (Karakasis et al., 2025b; Lam et al., 2022; Plana et al., 2022).

### 4.1 Virtual population development

#### 4.1.1 Necessity of allometric scaling

As described in section 2.2.4, allometric scaling of the DREAM population posterior was required to more fully match target measurement variability, particularly in the case of ventricular end-diastolic and stroke volumes. The lack of variability in the preliminary population posterior in these specific areas may be in part a consequence of constraining multiple non-independent measurements in the composite likelihood used to estimate this population posterior. Specifically, ejection fraction can be computed from the end-diastolic and stroke volume for a particular cardiac chamber, and the RV to LV end-diastolic volume ratio can be computed from the two individual chamber volumes; therefore, these are clearly not independent sets of measurement observations. Nevertheless, as we did not have information on the covariance of these quantities in the underlying population, we assumed independence in computing a simple composite marginal likelihood as the product of each one-dimensional measurement likelihood (Varin et al., 2011).

It is important to note that the reported literature means for these non-independent quantities were internally consistent; in other words, the mean ejection fraction is consistent with the computation from the corresponding mean end-diastolic and stroke volume for each chamber, as is the ventricular volume ratio (Petersen et al., 2017; McCracken et al., 2023). This allowed us to achieve a single RVI parameter set consistent with the mean phenotype. Yet while the mean measurement data were internally consistent, and therefore redundant, the corresponding standard deviations provided unique information. Depending on how individual end-diastolic and stroke volumes are paired within the patients making up the study sample, the same variability in volumes could lead to different variability in ejection fractions and the ventricular volume ratio. In fact, when we approximated a population posterior without using the ejection fraction and volume ratio measurements, we found that those measurements took on a wide range of unphysiological values in the resulting posterior measurement distributions (see **Supplementary Figure 4**). Therefore, we chose to explicitly consider all measurements, despite the risk that this could overconstrain the population likelihood in the area of chamber volumes. To mitigate this risk, we subsequently utilized allometric scaling as a physiology-based mathematical tool to increase variability in volume measurements without significantly changing pressure distributions (Günther, 1975), which were not subject to such dependent measurement data and were therefore less likely to observe systematic underestimation of variability.

#### 4.1.2 Variability in the final virtual population

Even after allometric scaling, our results show that matches of our goal of 80% to 110% of the target variability could only be obtained for 17 of 19 measurements, with the remaining two measurements capturing between 74% and 80% of the target variability. While this represents a promising result, covering the majority of all target measurement distributions, it also suggests areas for further consideration.

An important concern when using literature data to build the Bayesian likelihood is data consistency. In constructing our healthy virtual population, we incorporated data from multiple clinical studies. Although large sample sizes helped ensure the representativeness of measurement values, differences between study populations can lead to inconsistencies between these data, meaning the model may not be able to adequately capture the data for any prior space. This issue is especially pronounced when fitting variances. For instance, if the data sets originate from populations with disparate levels of variability on different measurements, measurements from populations with lower variability may artificially constrain the variability in other measurements.

The model parameters must also be considered in this matter. While our global sensitivity analysis aimed to find the most important parameter axes for variability in the virtual population, it did not account for the fact that, due to the interconnected nature of our model, multiple measurements may be highly sensitive to the same parameter. For example, the parameter SBV0 has global impacts throughout the model, with 15 of 19 simulation outputs having a Sobol index of at least 0.1 for this parameter (see **Supplementary Figure 2**). In the case of our simulation outputs with the lowest percent coverage of the target healthy population distribution, systolic and diastolic pulmonary arterial pressures, the most sensitive parameter for each of these outputs was also the largest contributor to the variability of at least two other measurements. Specifically, LV_EDV7.5, the most sensitive parameter for diastolic pulmonary arterial pressure, had the largest Sobol index for seven other output measurements, while RV_EDV5, the most sensitive parameter for systolic pulmonary arterial pressure, had the largest Sobol index for two other measurements. Therefore, these parameter distributions may have been constrained by other even more sensitive measurements, not allowing them to contribute sufficiently to the variability on pulmonary arterial pressures.

Given the current limitations in virtual population coverage of pulmonary vascular measurements, downstream digital twin development may require special handling such as an exclusion criteria that prevents or limits digital twin applicability when patient observations for these measurements fall outside of the covered ranges. Nevertheless, the results demonstrate that a substantial portion of the target population variability could be achieved, and further work may best be guided by study of the virtual population’s ability to match individual patient clinical data, as will be discussed in the next section.

### 4.2 Patient-specific digital twins

Numerous Bayesian techniques have been developed to estimate parameter distributions that reproduce target patient-specific measurement distributions, including methods such as approximate Bayesian computation, adaptive importance sampling, and Markov chain Monte Carlo methods (Csilléry et al., 2010; van Osta et al., 2021; Santos and Valero-Cuevas, 2004; Rannala, 2002; Schiavazzi et al., 2015). However, these approaches typically estimate a single patient-specific posterior distribution by adaptively sampling and simulating parameter sets during inference; as a result, these techniques require potentially time-consuming simulations to be run separately for each patient.

We applied one such Bayesian technique, the DREAM algorithm (Vrugt et al., 2009), in our virtual population development methodology, yet we chose a different approach for patient-specific digital twins. The methodology presented in section 2.3 uses the precomputed virtual population as a fixed prior distribution from which to select patient-specific digital twins. While the virtual population is designed to cover the expected phenotypic variability of the underlying patient population of interest, the use of actual patient-specific measurements introduces additional considerations as discussed below.

#### 4.2.1 Matching of pulmonary pressures

In the virtual population, only systolic and diastolic pulmonary arterial pressure failed to achieve at least 80% of the target coverage. Unsurprisingly, the digital twin results similarly show their weakest performance in matching of pulmonary pressures, with all four cases of the target measurement value falling outside of the 5th to 95th percentile of the posterior distribution occurring for a pulmonary pressure measurement (see **Figure 5**). The twin with the highest MAPE (Patient 4 in **Figure 7**) additionally shows clear difficulty matching the target pulmonary pressures, with a significant underestimation of systolic pressure and overestimation of diastolic pressure. This hints additionally at the importance of not only coverage of marginal measurement distributions, but also their joint distributions, as will be discussed in the following section.

#### 4.2.2 Coverage of joint measurement distributions

Even if the virtual population perfectly matches each target marginal measurement distribution, this does not guarantee joint coverage of all possible patient-specific combinations. For example, even if the marginal distributions of both systolic and diastolic pressures are matched, we may see that the pairing of a high systolic pressure with a low diastolic pressure is not adequately represented; the digital twin of Patient 2 in **Figure 7** is illustrative, with a slightly lower systolic aortic pressure distribution peak compared to the observed value, despite apparent population-level coverage of the target pressure. The digital twin of Patient 4 shows a more dramatic example where a large pulmonary arterial pulse pressure is not adequately matched in the posterior distribution; this is likely a result of both a shortcoming in matching marginal distributions for these measurements and further lacking coverage in the joint distribution.

In order to ensure that all such combinations are matched, it is necessary to utilize a large dataset of linked patient-specific measurements, or at a minimum, detailed characterization of two-dimensional and higher joint distributions on pairs and combinations of measurements at the population level. It may also require that the parameter space for the virtual population be determined on the basis of not only individual measurements, but also on their relationships. For example, one could repeat the global sensitivity analysis of section 2.2.2 while also considering key relationships between measurements; we already considered some such aspects by incorporating ejection fractions and the ventricular end-diastolic volume ratio, but further additions such as aortic and pulmonary arterial pulse pressures may help to further improve population-level coverage of the linked measurement data that occurs in individual patients.

#### 4.2.3 Influence of the virtual population prior

A key aspect of Bayesian inference is the incorporation of prior knowledge. This prior knowledge can range from uninformative, such as broad uniform distributions across all parameters, to highly informative, as in the case of our virtual population. In addition to the computational benefit provided by our precomputed prior approach, an informative prior can improve parameter identifiability even for an overparameterized model (Rannala, 2002). In the case of our model, where the parameters to vary were chosen on the basis of global sensitivity analysis for the set of hemodynamic data available at the population level, the model becomes overparameterized when faced with the case of a more limited set of data available for an individual patient in a realistic clinical setting. The virtual population prior ensures that, in the absence of patient-specific measurement observations, simulation results are still constrained to fall within the physiological space of the broader population.

To demonstrate this behavior, we also estimated four patient-specific posterior distributions (digital twins) directly through the DREAM algorithm using only the available patient-specific measurement data to construct the likelihood (see **Supplementary Section 2.4.3**). As expected, the resulting posterior distributions have well-constrained simulation outputs for those quantities that correspond to available clinical measurements, similar to the original digital twins from section 3.2. However, other distributions without available patient data took on unphysiological values, such as very large ranges of LV ejection fraction and the ventricular end-diastolic volume ratio (see **Supplementary Figure 10**).

As this example illustrates, the virtual population prior beneficially enforces reasonable physiologic distributions where patient-specific measurements are unavailable. However, it does additionally serve to bias digital twin posterior distributions towards the population-level mean, even in cases where patient-specific data are available; for example, we see results suggesting this behavior in the the distribution of diastolic pulmonary arterial pressure for Patient 3 in **Figure 7**. If the virtual population prior is sufficiently broad relative to the patient-specific likelihood, this behavior is minimized; locally, a wide Gaussian distribution is relatively flat. Additionally, even uniform priors can have unexpected consequences on posterior distributions (Dongen, 2006), so we believe that the benefits of the virtual population prior outweigh the disadvantages and that a small bias towards the population prior will not significantly impact patient-specific posterior distributions. However, awareness of this tradeoff between informative and uninformative prior knowledge is critical so that it can be evaluated within the context of relevant clinical use cases.

### 4.3 Strengths and limitations

This work presented a methodology to develop a virtual population, a collection of parameter sets that reproduces the phenotypic variability seen in a real population of interest. We demonstrated this method on a lumped-parameter hemodynamic model to develop a healthy virtual population which provided at least 74% of the expected coverage of the target distributions of 19 pressure and volume measurements from the literature on healthy populations, with 17 of 19 measurements having between 80% and 110% coverage. We additionally demonstrated feasibility in a proof-of-concept use of this virtual population as the fixed, precomputed prior for patient-specific Bayesian inference for 17 patients without noted cardiovascular disease.

By creating a virtual population spanning the phenotypic variability of the true population of interest up front, the scalability and computational efficiency of obtaining patient-specific digital twins could be improved, addressing a key barrier to the practical adoption of digital twins in medicine (Peirlinck et al., 2021; Coorey et al., 2022; Viceconti et al., 2024). A precomputed virtual population supports a full understanding of the variability within the underlying system in advance; the simulations required for any individual patient have already been run and can be rapidly identified through Bayesian inference within that preexisting prior distribution. For the results presented here, holding software and hardware capabilities constant, performing the computations to obtain patient-specific digital twins from the precomputed virtual population prior took less than two seconds, while using the DREAM algorithm to perform more traditional patient-specific Bayesian inference required at least 12 hours apiece. While the upfront investment to create a virtual population is non-trivial (approximately 78 hours for the results presented here, as detailed in the **Supplementary Material**), this investment can pay off even for small numbers of patients, with a break-even point of less than seven patients for the present case. By starting from a virtual population, the computational complexity of obtaining patient-specific digital twins no longer scales with the number of patients being treated, helping remove one barrier to wide-scale adoption of this technology. While other methods have been suggested using emulators or machine learning surrogates to improve computational efficiency, these methods often require a cohort of individual patient digital twins as an input to the process (Lanyon et al., 2026; Romero et al., 2021), whereas our method can start from published population-level data from the literature.

While this paper has focused on the applications of a virtual population towards patient-specific digital twins, a virtual population may also be of use in future applications such as virtual clinical trials, supporting *in silico* evaluation of new treatments and devices (Chase et al., 2018; Dasí et al., 2024; Gillette et al., 2023). As regulatory agencies encourage the use of new approach methodologies (NAMs) to reduce the animal burden of preclinical testing wherever possible, such *in silico* testing becomes increasingly valuable and recognized as an important aspect of any regulatory strategy (Sewell et al., 2024; FDA, 2025).

Nevertheless, the work presented here leaves several limitations to be considered in future studies. First, as mentioned previously, matching one-dimensional or marginal measurement distributions does not guarantee that we adequately represent the joint multi-dimensional measurement distributions observed across individual patients. Validation of the virtual population against larger sets of patient-specific data, and likely refinements to the virtual population on the basis of those results, will still be required. Further, the set of MIMIC-IV patients used for patient-specific digital twins in the present work does not constitute a representative cohort of healthy volunteers; as patients were still hospitalized after a liver transplant, they may have experienced mechanical ventilation, various pharmaceutical interventions, systemic inflammation, and other post-surgical effects that could materially impact hemodynamics. Additionally, the available patients satisfying the selection criteria skewed heavily male (15 of 17 patients, see **Supplementary Table 4**). Future work should seek to find a larger and more representative healthy cohort, and validation must encompass not only the virtual population’s ability to create digital twins representative of patients in a single physiological state, but also the ability of those twins to predict patient responses to different scenarios, such as drug and device treatments or physical exertion.

The heterogeneity of the study populations used as measurement targets also introduces uncertainties in the compatibility of those data sets. By incorporating different underlying clinical populations which all exist within the broader healthy population, we have attempted to provide as much coverage of that true population as possible; the variability between the populations under study should necessarily be less than the variability within the full target population, and thus can still be viewed as a representation of clinically-meaningful variability that our virtual population should represent. However, future work would benefit from a larger, unified population with data not only for marginal measurement distributions, but also describing the covariance amongst different measurements. Further benefits could be derived from constructing more targeted virtual subpopulations stratified on characteristics such as sex, age, and body size. Such virtual subpopulations could be used to better understand differences between corresponding segments of the real population, as well as to support a more informative prior for each patient on the basis of such characteristics.

This work focused on a healthy population, which limits the applicability of treatment predictions. This choice was made to provide a strong baseline against which future cardiovascular disease populations could be compared and better contextualized, and because there are large sample sizes of healthy, stable patients available in the literature. However, to develop clinically-meaningful digital twins to facilitate treatment of cardiovascular disease, virtual populations covering these disease states are of course required. Future work must therefore expand the concepts presented here to cover relevant disease populations, which will likely require consideration not only of a wider range of parameter values, but also of additional model parameters, such as parameters characterizing the cardiac valves to develop a virtual population for valvular diseases. Nevertheless, the approaches presented here should provide a strong baseline to facilitate handling these new cases in the future.

Finally, the preliminary digital twins as presented in this work do not yet explicitly cover certain key aspects of a true digital twin, as defined by the National Academies of Science, Engineering, and Medicine: dynamic updating, predictive capabilities, and the ability to inform decision-making (National Academies of Sciences, Engineering, and Medicine, 2024). However, in future work, each of these expansions could readily fit within the framework presented here. Depending on the time frame, dynamic updating could be performed in a variety of ways; for example, in the case where more diagnostic data become available throughout a patient’s hospital stay, the virtual population prior could subsequently be replaced by an earlier digital twin posterior distribution, using one day’s posterior as the next day’s prior, further constraining the updated digital twin on the basis of new available data. Predictive capabilities and the ability to inform decision-making are similarly compatible within the present Bayesian inference framework, where a “baseline” digital twin could be subsequently simulated under different pharmaceutical and device treatments, modeled in terms of their impact on existing model parameters (Kataoka et al., 2026; Matsushita et al., 2025).

### 4.4 Conclusions

In this work, we presented a methodology to establish a virtual population from model-connected measurement data of a population of interest and used the resulting virtual population as a fixed, precomputed prior for efficient patient-specific Bayesian inference to determine probabilistic digital twins. While the present work created a healthy virtual population as a baseline, using the published hemodynamic data of healthy patient populations, other virtual populations can be developed under the same approach to support mechanistic insights into different pathophysiological conditions and disease states. Ultimately, these virtual populations and the patient-specific digital twins they enable may support improved patient phenotyping and clinical decision-making in the treatment of cardiovascular disease.

## Data Availability

Publicly available datasets were analyzed in this study. This data can be found here: https://physionet.org/content/mimiciv/3.1/ and the article’s **Supplementary Material**.
