# Supplementary material for: Accelerating cardiovascular digital twin creation using precomputed virtual populations as Bayesian priors
Source: Front Physiol. 2026 Sep 21;17:1950271. doi: 10.3389/fphys.2026.1950271 (PMC13658946; doi:10.3389/fphys.2026.1950271)
Supplement: Supplementary file 1 [file SupplementaryFile1.pdf]

frontiers

Supplementary Material

1 SUPPLEMENTAL METHODS

1.1 Lumped-parameter hemodynamic model

1.1.1 Time-varying elastance computations

Within the lumped-parameter cardiovascular model, each chamber's instantaneous pressure is approximated as the sum of active (systolic) and passive (diastolic) components:

$$P(t) = P_{es}(t) + P_{ed}(t) \tag{S1}$$

where $P_{es}(t)$ represents the active or systolic portion of pressure and $P_{ed}(t)$ represents the passive or diastolic portion of pressure (Shimizu et al., 2018; Suga et al., 1973; Burkhoff et al., 1988; Alexander Jr et al., 2018; Stergiopulos et al., 1996).

The systolic component $P_{es}(t)$ depends upon instantaneous chamber volume $V(t)$ and a time-varying elastance $E(t)$:

$$P_{es}(t) = E(t) \cdot V(t) \tag{S2}$$

The time-varying elastance is defined as follows for each cardiac cycle:

$$E(t) = \begin{cases} \frac{\text{Ees}}{2}\left[\sin\left(\frac{\pi t}{T_{max}} - \frac{\pi}{2}\right) + 1\right], & \text{if } t < T_{max} \\ \frac{\text{Ees}}{2}\left[\cos\left(\frac{t - T_{max}}{\tau}\right) + 1\right], & \text{if } T_{max} \leq t < T_{max} + \frac{\pi\tau}{2} \\ \frac{\text{Ees}}{2} \cdot e^{\left(T_{max} + \frac{\pi\tau}{2} - t\right)/\tau}, & t \geq T_{max} + \frac{\pi\tau}{2} \end{cases} \tag{S3}$$

where t is relative to the cardiac cycle, so it resets to 0 seconds upon the start of each beat. In this piecewise continuous formulation, elastance is approximated as a sinusoidal increase to a peak of the chamber-specific Ees (LA_Ees, LV_Ees, RA_Ees, or RV_Ees) at time T_{max}, after which relaxation begins, similar to several previously-published works (Shimizu et al., 2018; Suga et al., 1973; Burkhoff et al., 1988; Alexander Jr et al., 2018). Relaxation is first sinusoidal and then exponential, with the time constant of relaxation defined as τ. The period of the sinusoid, the timing of the transition, and the amplitudes of the segments are matched such that relaxation transitions smoothly between the two portions. The timing parameters T_{max} and τ are calculated from heart rate, which is set according to measured values in the population-level literature. As a result, the timing parameters (both with units of seconds) can be treated as chamber-specific fixed parameter values, calculated to produce a physiologically reasonable time course depending on the current heart rate (HR, in units of beats per minute):

- Left and right ventricle: $T_{max} = 0.522 - 0.123 \cdot \text{HR}/60$ and $\tau = T_{max}/8$
- Left and right atrium: $T_{max} = 0.145 - 0.018 \cdot \text{HR}/60$ and $\tau = T_{max}/4$

Additionally, the timing of the atria and the ventricles are offset by a heart rate-dependent delay, calculated in seconds as $0.18 - 0.00035 \cdot \text{HR}$ to mimic a typical healthy PR interval (Carruthers et al., 1987).

The passive component of pressure P_{ed} is computed based on a single-point estimate of the pressure-volume curve, as developed by Klotz et al. (2006):

$$P_{ed}(t) = \alpha V(t)^{\beta} \tag{S4}$$

where the parameters α and β are calculated as follows:

$$\alpha = 30/V_{30}^{\beta} \tag{S5}$$

$$\beta = \log_{10}\left(\frac{\mathrm{EDP}}{30}\right) / \log_{10}\left(\frac{\mathrm{EDV}}{V_{30}}\right) \tag{S6}$$

Within these equations, V_0 and V_{30} are computed from the chamber-specific end-diastolic pressure and volume point parameters, EDP and EDV (which correspond to the pressure-volume point denoted (P_m, V_m) in the original equations of Klotz et al.), along with constants $A_n = 27.78$ and $B_n = 2.76$ (Klotz et al., 2006):

$$V_0 = \mathrm{EDV} \cdot (0.6 - 0.006 \cdot \mathrm{EDP}) \tag{S7}$$

$$V_{30} = V_0 + (\mathrm{EDV} - V_0) \,/\, (\mathrm{EDP}/A_n)^{(1/B_n)} \tag{S8}$$

The pressure point parameter (EDP) for each chamber was fixed according to average normal end-diastolic pressures (Klingensmith et al., 2012), with the corresponding volume point (EDV) being treated as the unknown parameter, where $\mathrm{LA\_EDV8.5}$ represents the LA end-diastolic volume point at a pressure of 8.5 mmHg, $\mathrm{LV\_EDV7.5}$ represents the LV end-diastolic volume point at a pressure of 7.5 mmHg, and $\mathrm{RA\_EDV5}$ and $\mathrm{RV\_EDV5}$ represent the RA and RV end-diastolic volume points, respectively, at a pressure of 5 mmHg.

In this way, time-varying pressure for each of the four cardiac chambers can be computed according to equation S1 and the supporting equations S2 – S8, where the only unknown parameters to be estimated are the four chamber-specific Ees and EDV values.

1.1.2 Cardiac valves

The atrio-ventricular and semi-lunar valves are modeled with diodes and resistors (Kaye et al., 2021; Bjørdalsbakke et al., 2022), with the possibility of both antegrade (forward) and retrograde (backwards) flow, resulting in two resistors for each valve: the mitral valve (MV, with $\mathrm{R_{VMV}}$ and $\mathrm{R_{VMR}}$ for forward and retrograde resistance, respectively), the aortic valve (AV, with $\mathrm{R_{VAV}}$ and $\mathrm{R_{VAR}}$), the tricuspid valve (TV, with $\mathrm{R_{VTV}}$ and $\mathrm{R_{VTR}}$), and the pulmonary valve (PV, with $\mathrm{R_{VPV}}$ and $\mathrm{R_{VPR}}$). Note that estimation of these parameter values was excluded from this investigation because they are not applicable to simulation of a healthy population. For this population with no significant valvular regurgitation or stenosis, the forward resistances ($\mathrm{R_{VMV}}$, $\mathrm{R_{VAV}}$, $\mathrm{R_{VTV}}$, and $\mathrm{R_{VPV}}$) were set to very low values (0.005 $\mathrm{mmHg \cdot s/mL}$) to allow unimpeded forward flow, while the four retrograde resistances ($\mathrm{R_{VMR}}$, $\mathrm{R_{VAR}}$, $\mathrm{R_{VTR}}$, and $\mathrm{R_{VPR}}$) were set to very high values (1000 $\mathrm{mmHg \cdot s/mL}$) to prevent backwards flow.

1.1.3 Vasculature

Modified Windkessel vascular components are used to represent the systemic and pulmonary vasculature (Kaye et al., 2021; Burkhoff et al., 1988; Stergiopulos et al., 1996; Westerhof et al., 2009), with each including a characteristic impedance ($\mathrm{Z_S}$ and $\mathrm{Z_P}$ for the systemic and pulmonary components, respectively), along with resistance and capacitance distributed between the arterial, capillary, and venous portions of the

vasculature: R_{SA}, R_{SC}, and R_{SV} as the systemic arterial, capillary, and venous resistances; C_{SA}, C_{SC}, and C_{SV} as the respective systemic compliances; R_{PA}, R_{PC}, and R_{PV} as the pulmonary resistances; and C_{PA}, C_{PC}, and C_{PV} as the corresponding pulmonary compliances. Note that, as detailed in the main body of the manuscript, the three pulmonary resistances and three pulmonary compliances were linked and varied together in this investigation due to lack of measurement data within this portion of the vasculature.

1.1.4 Stressed blood volume

Finally, an initial stressed blood volume ($SBV0$) is distributed throughout the heart and the stressed vasculature (Shimizu et al., 2018; Burkhoff and Tyberg, 1993); in the electrical analogue representation, this is an initial charge distributed amongst the capacitors of the four heart chambers and the vasculature.

1.1.5 Initial parameter values and range limits

Initial parameter values and range limits, if applicable, for each of the 23 relevant unknown model parameters are provided in Table S1. Range limits for the EDV parameters are set based on the population-level measurement data from Petersen et al. (2017), specifically ranging from two standard deviations below to two standard deviations above the population-level mean. Other ranges are chosen to be as broad as possible while maintaining mathematically and physiologically reasonable simulation results.

Table S1. Parameters for inference, including units, initial values, and range limits. Note that C_{PC} and C_{PV} are linked to C_{PA}, and R_{PC} and R_{PV} are similarly linked to R_{PA}, varying each set of three parameters together while maintaining the same ratio as their initial values; as such, their range limits are also linked in those same ratios.

**Parameter**	**Units**	**Initial Value**	**Range Limits**
C_{PA}	$mL/mmHg$	6.42	0.1 – 25
C_{PC}	$mL/mmHg$	6.64	0.103 – 25.9
C_{PV}	$mL/mmHg$	6.88	0.107 – 26.8
C_{SA}	$mL/mmHg$	1.57	0.1 – 10
C_{SC}	$mL/mmHg$	6.62	0.1 – 20
C_{SV}	$mL/mmHg$	125	10 – 500
LA_EDV8.5	mL	66.0	28 – 104
LA_Ees	$mmHg/mL$	0.212	0.01 – 1
LV_EDV7.5	mL	143	75 – 211
LV_Ees	$mmHg/mL$	2.10	0.5 – 5
RA_EDV5	mL	80.0	30 – 130
RA_Ees	$mmHg/mL$	0.189	0.01 – 1
RV_EDV5	mL	154	74 – 234
RV_Ees	$mmHg/mL$	0.364	0.1 – 1
R_{PA}	$mmHg \cdot s/mL$	0.0204	0.005 – 0.25
R_{PC}	$mmHg \cdot s/mL$	0.0511	0.0125 – 0.625
R_{PV}	$mmHg \cdot s/mL$	0.0283	0.00691 – 0.346
R_{SA}	$mmHg \cdot s/mL$	0.806	0.005 – 2
R_{SC}	$mmHg \cdot s/mL$	0.161	0.005 – 1
R_{SV}	$mmHg \cdot s/mL$	0.0540	0.005 – 1
SBV0	mL	1860	500 – 5000
Z_P	$mmHg \cdot s/mL$	0.0250	0.005 – 0.25
Z_S	$mmHg \cdot s/mL$	0.0510	0.005 – 1

Note that the initial value for SBV0, which remained fixed during parameter point estimates and is therefore the only initial value used directly in subsequent steps of our methodology, was computed from the measured values reported in Fudim et al. (2022) plus the sum of the four reported chamber mean end-diastolic volumes of Petersen et al. (2017); this adjustment reflects our model implementation where SBV0 is the blood volume distributed amongst not only the stressed vasculature but also the heart.

1.2 Measurement targets from the literature

Measurement targets from the published literature on healthy populations, corresponding to 20 model outputs, are provided in Table S2. Note that mean mid-capillary pressure is only used for determination of the healthy reference virtual individual (RVI) parameter point estimate, as described in the manuscript section 2.1.2; it is not used as a target for variability matching in the virtual population.

Table S2. Measurement data from the literature.

Measurement	Units	Mean	Standard Deviation
Left atrial end-diastolic volume (Petersen et al., 2017)	mL	66	19
Left atrial stroke volume (Petersen et al., 2017)	mL	40	11
Left atrial ejection fraction (Petersen et al., 2017)	%	60	7
Right atrial end-diastolic volume (Petersen et al., 2017)	mL	80	25
Right atrial stroke volume (Petersen et al., 2017)	mL	35	13
Right atrial ejection fraction (Petersen et al., 2017)	%	44	10
Left ventricular end-diastolic volume (Petersen et al., 2017)	mL	143	34
Left ventricular stroke volume (Petersen et al., 2017)	mL	85	20
Left ventricular ejection fraction (Petersen et al., 2017)	%	60	6
Right ventricular end-diastolic volume (Petersen et al., 2017)	mL	154	40
Right ventricular stroke volume (Petersen et al., 2017)	mL	85	20
Right ventricular ejection fraction (Petersen et al., 2017)	%	56	6
Right-to-left ventricular end-diastolic volume ratio (McCracken et al., 2023)	unitless	1.07	0.11
Systolic aortic pressure (Wright et al., 2011)	mmHg	117	11
Diastolic aortic pressure (Wright et al., 2011)	mmHg	69	9.6
Systolic pulmonary arterial pressure (Kovacs et al., 2009)	mmHg	20.8	4.4
Diastolic pulmonary arterial pressure (Kovacs et al., 2009)	mmHg	8.8	3
Mean left atrial pressure (Kovacs et al., 2009)	mmHg	8.0	2.9
Mean right atrial pressure (Reddy et al., 2019)	mmHg	5	2
Mean mid-capillary pressure (Morissette, 1977)	mmHg	20.5	4.3

1.3 Parameter point estimates

In combination with the normalized root mean square error (NRMSE) to quantify agreement between simulation outputs and the measurement targets, the parameter fitting cost function incorporated a penalty function to enforce parameter ranges. This penalty function was formulated to increase quadratically from a value of 0 within the allowed ranges to a value of 100 at a normalized distance of 1 outside of the ranges. The normalization factor is the distance from the lower to upper bound or, when the lower bound is near zero, the distance from the lower bound to zero:

$$\mathrm{Penalty}\,(\theta_i) = \begin{cases} 0, \text{ if } LB_i \leq \theta_i \leq UB_i \\ 100 \cdot \left(\frac{LB_i - \theta_i}{min(LB_i, UB_i - LB_i)}\right)^2, \text{ if } \theta_i < LB_i \\ 100 \cdot \left(\frac{\theta_i - UB_i}{UB_i - LB_i}\right)^2, \text{ if } \theta_i > UB_i \end{cases} \tag{S9}$$

where LB_i and UB_i represent the parameter-specific lower and upper bounds and θ_i is the value of the parameter θ. The overall parameter range penalty is the sum of the individual $\mathrm{Penalty}$ values across all $N = 18$ parameters.

An iterative fitting approach was used in which parameter ranges were repeatedly reduced to obtain higher precision estimates. At the first round of fitting, parameter ranges were as detailed in Table S1. Within these ranges, 50 parameter fits were performed, with starting values set according to Latin Hypercube Sampling (LHS) (Loh, 1996) within ranges of 0.5 to 1.5 times the initial values listed in Table S1.

Subsequently, the best 10 of 50 parameter fits, in terms of minimized cost function, were used to restrict search ranges for the next round of fitting, specifically setting the allowed range to the previous fitted parameter range plus a buffer of 5% of that range on both ends. Starting values for the next iteration of parameter fits were set according to LHS within these search ranges. This process was repeated, adjusting the search ranges at each iteration, until all parameter ranges converged to less than 1% of their corresponding initial range. At this point, the mean of the top 10 final fits was taken as the reference virtual individual (RVI) parameter set.

1.4 MIMIC-IV patient selection criteria

Patients were selected from the MIMIC-IV database (Johnson et al., 2023, 2024) based on the following criteria:

1. Systolic and diastolic aortic pressure (item IDs 220050 and 220051), systolic and diastolic pulmonary artery pressure (item IDs 220059 and 220060), central venous pressure (item ID 220074), cardiac output (item ID 220088, 224842, or 228369), and heart rate (item ID 220045) were all present within the same one-hour period.
2. Each of the above measurements were within one standard deviation of the population level mean from Table S2. Note that cardiac output and heart rate were used to compute stroke volume for these purposes because cardiac output was significantly more available within the MIMIC-IV population than stroke volume.
3. The patient's purpose of hospitalization was liver transplant (ICD-9 procedure code of 5051, 5052, 5053, 5054, or 5059, or ICD-10 code of 0FY00Z0, 0FY00Z1, or 0FY00Z2).

Liver transplant was chosen as the most common non-cardiac cause of hospitalization amongst the 96 unique patients meeting the first two criteria. All other common procedures were cardiovascular in nature:

cardiac valve replacement or repair (n=54), coronary artery bypass (n=24, including 5 who also had a valve-related procedure), and thoracic aorta or other vessel replacement (n=5).

In the case that a patient had multiple one-hour periods satisfying all selection criteria, the earliest qualifying time point was selected to maintain a cohort of unique patients.

2 SUPPLEMENTAL RESULTS

2.1 Parameter point estimates

The iterative parameter fitting process to determine a single RVI parameter set consistent with the target mean phenotype required 12 rounds to satisfy the convergence criteria, as depicted in Figure S1. The figure's y-axis limits are set to the search range for each parameter at the initial fitting round, highlighting that those ranges are not overconstraining the search space; except in cases involving the natural lower bound near zero, the fitted values are not approaching the range limits.

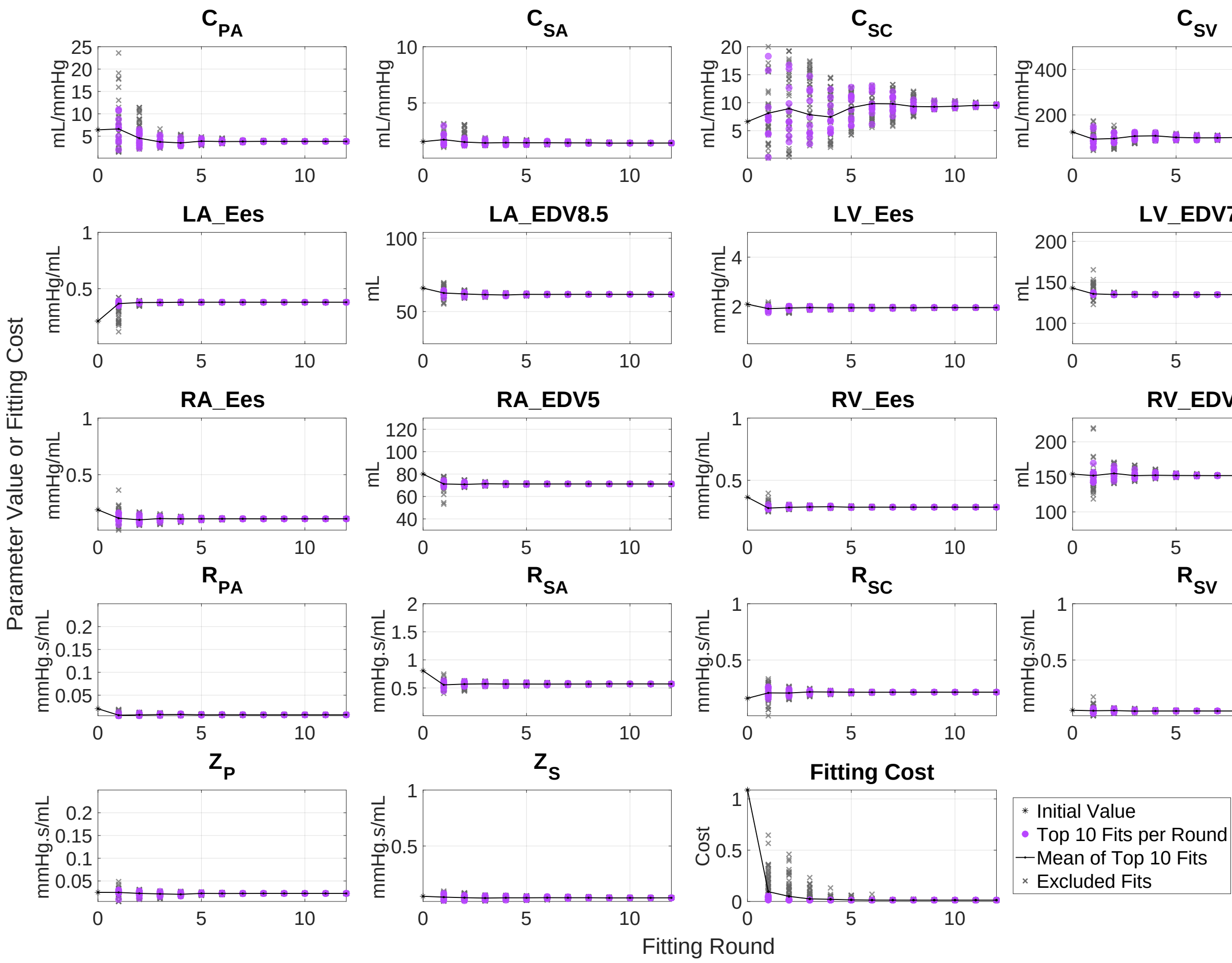

Figure S1. Parameter point estimates and corresponding cost function value before fitting (round 0) and across iterative fitting rounds 1 through 12. The top 10 fits at each round in terms of cost function minimization are displayed in purple, while the remaining 40 excluded fits are shown in grey. The black line highlights the mean of the top 10 fits and the corresponding cost function value for that mean parameter set. Note that y-axis limits are set to the search range at the initial fitting round, as defined in Table S1.

2.2 Global sensitivity analysis

Global sensitivity analysis results around the RVI parameter set found that 13 parameters had a total Sobol index above the sensitivity threshold of 0.05 for at least one measurement: C_{SA}, C_{SV}, LA_Ees, LA_EDV8.5, LV_Ees, LV_EDV7.5, RA_Ees, RA_EDV5, RV_Ees, RV_EDV5, R_{SA}, R_{SV}, and SBV0. The remaining six parameters (C_{PA}, C_{SC}, R_{PA}, R_{SC}, Z_P, and Z_S) had no Sobol indices above the threshold. Note that while there is no universally accepted threshold for parameter space reduction, a value of 0.05 is widely adopted in similar analyses (Zhang et al., 2015).

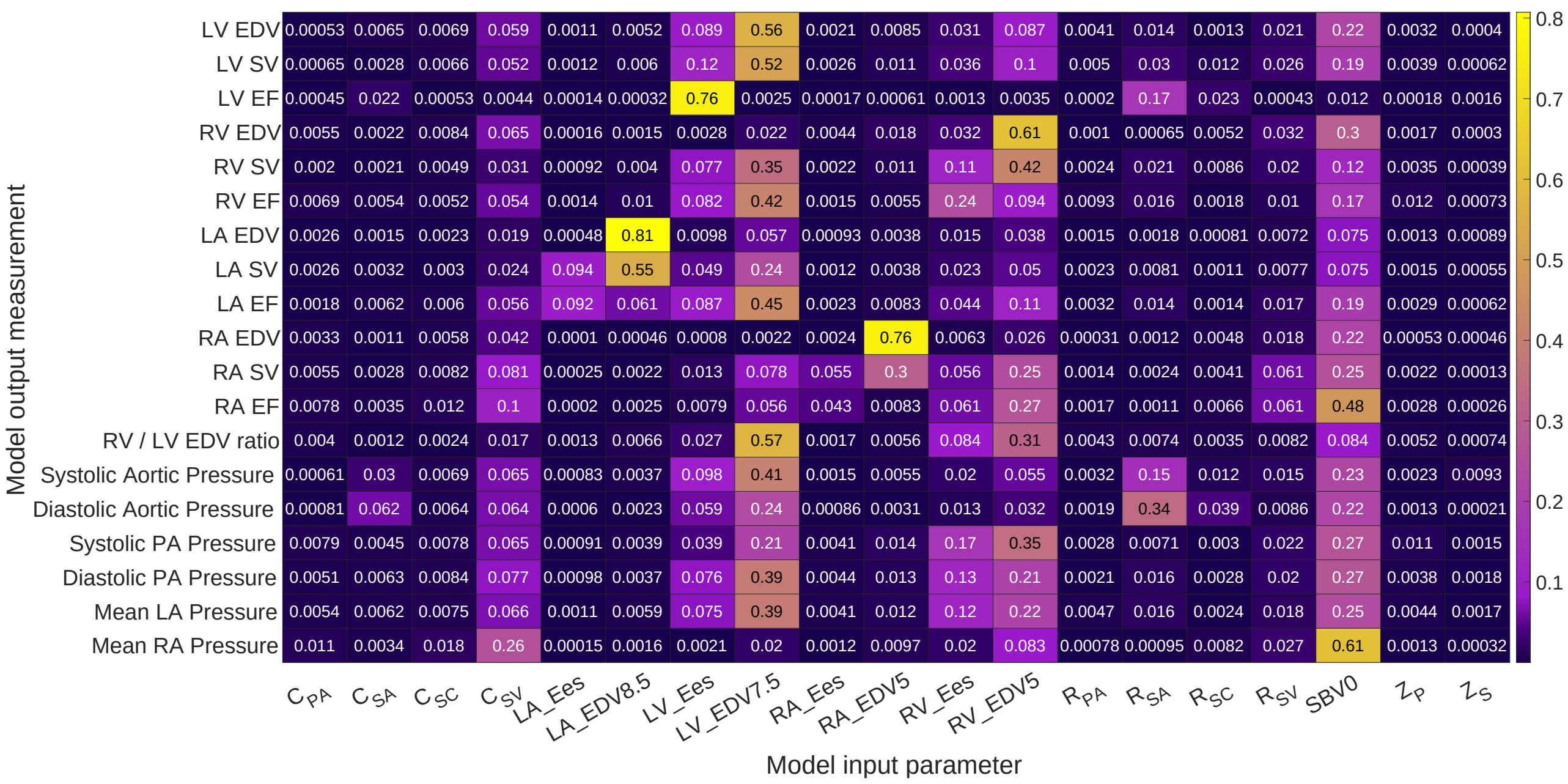

Figure S2. Total Sobol indices for all model parameters and relevant output measurements.

2.3 Population posterior

2.3.1 Impact of ejection fractions and volume ratio

As discussed in the manuscript, the likelihood used to estimate the population posterior is internally dependent, with end-diastolic volume, stroke volume, and ejection fraction for each cardiac chamber being directly algebraically related, as are the two ventricular end-diastolic volumes and the RV to LV end-diastolic volume ratio. One possible solution to this issue would be to keep only the minimal set of independent measurements, for example by excluding the four ejection fractions as well as the ventricular volume ratio.

To explore this possibility, we reran the DiffeRential Evolution Adaptive Metropolis (DREAM) algorithm (Vrugt et al., 2009; Vrugt, 2016) with this updated formulation of the likelihood (14 measurements instead of 19), with results displayed in Figures S3 (parameter marginal distributions) and S4 (measurement distributions). As illustrated in the plot of measurement distributions, exclusion of the ejection fractions and ventricular volume ratio resulted in those measurements expanding into unphysiological ranges in the second posterior. While coverage of ventricular end-diastolic and stroke volumes did improve slightly, as would be expected under this less-constrained configuration, the corresponding ejection fraction and

volume ratio distributions indicate that many of the virtual individuals making up this expansion are physiologically implausible in terms of the relationships between the fitted volumes. As a result, we chose to move forward using all 19 measurements in the likelihood, despite the internal dependence that this introduces.

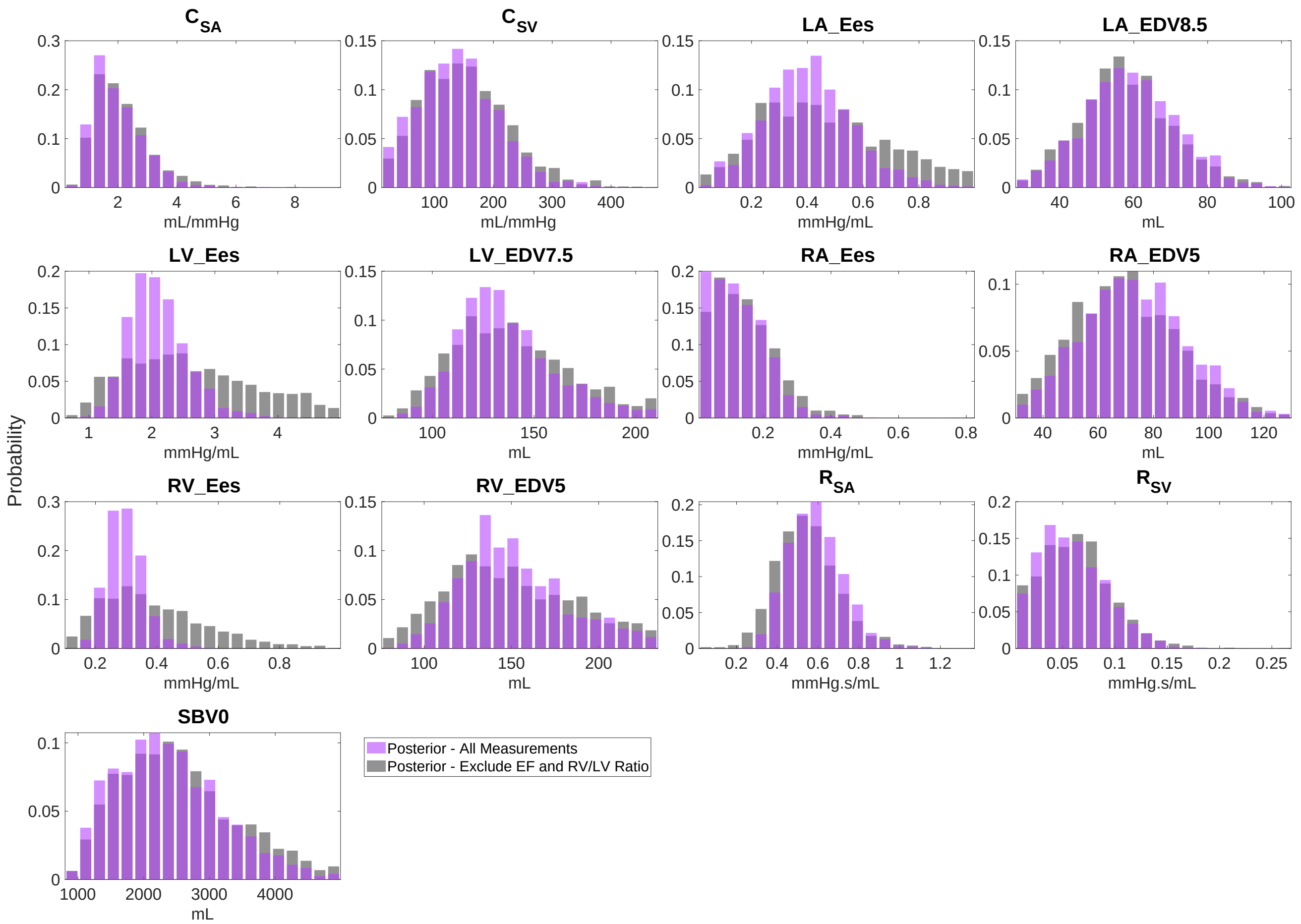

Figure S3. One-dimensional parameter marginal distributions for the population posterior obtained using all 19 measurements (purple) and for the posterior obtained using only 14 measurements, excluding ejection fractions and the ventricular volume ratio (grey).

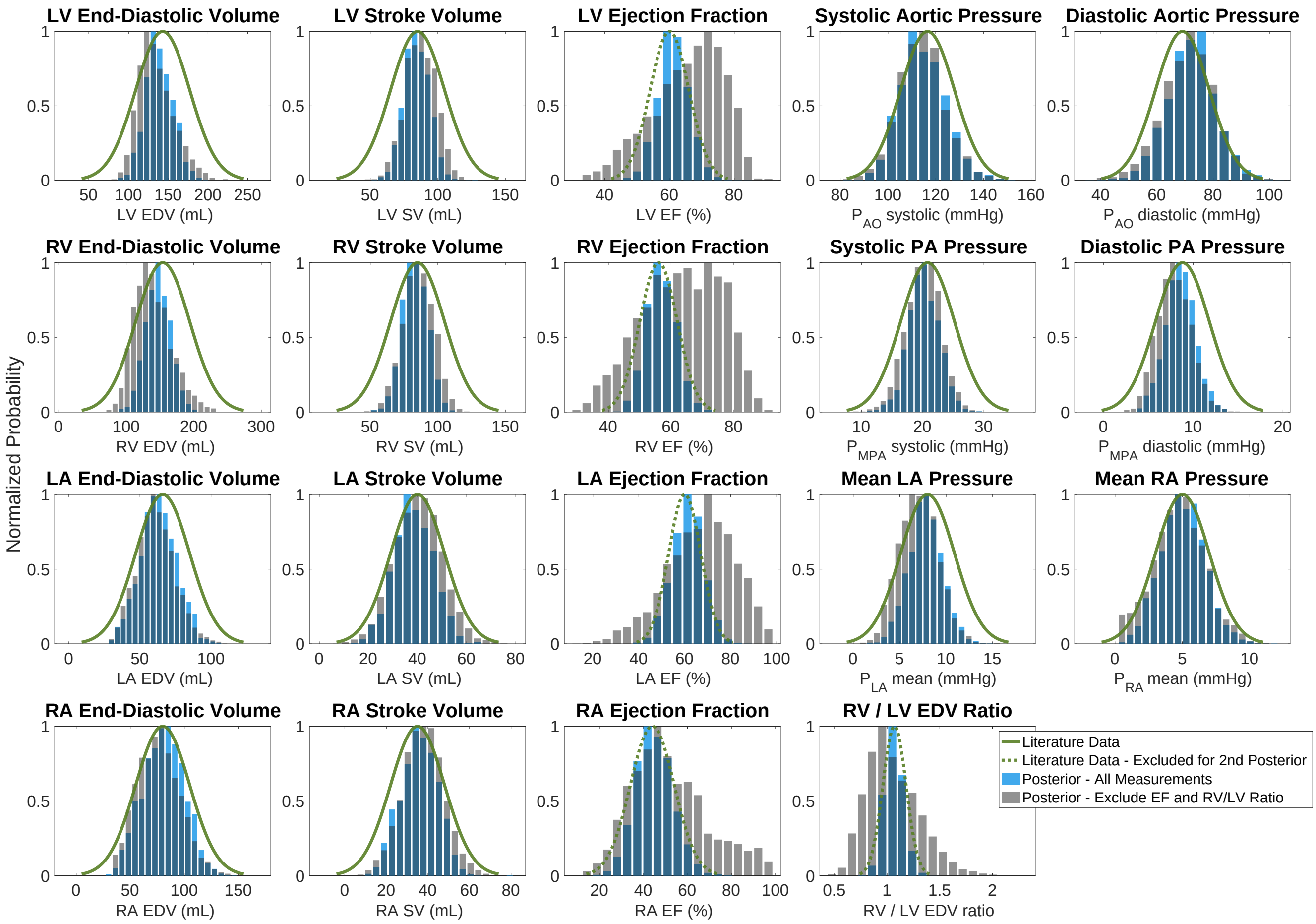

Figure S4. Model output measurement distributions corresponding to the population posterior obtained using all 19 measurements (blue) and the posterior obtained using only 14 measurements, excluding ejection fractions and the ventricular volume ratio (grey). Green Gaussian curves highlight the population-level measurement target data from the literature, with the ejection fraction and ventricular volume ratio data that were excluded from the second posterior's likelihood displayed with dashed lines. For ease of comparison, all histograms and Gaussian curves are normalized to have a consistent peak amplitude of 1.

2.3.2 Choice of prior sigma

In determining the population posterior, a lightly informative prior distribution was applied around the RVI parameter set, formulated as a Gaussian with both the mean and standard deviation for each parameter set to the corresponding RVI parameter value. This choice was made to encourage the posterior distribution to center near the RVI parameter set while still allowing significant exploration of other areas of the parameter space. However, other prior formulations could of course be chosen, particularly in relation to the value used for the prior's standard deviation.

To investigate the impact of such changes, we reran the DREAM algorithm under three different prior configurations: standard deviation equal to 0.5, 1, and 2 times the corresponding RVI parameter value. As would be expected, posterior parameter distributions tended to be narrower for the more constrained prior (standard deviation equal to half the RVI value) and wider for the less constrained prior (standard deviation equal to twice the RVI value), as shown in Figure S5. Interestingly, the corresponding measurement distributions exhibit relatively little change between these configurations, as shown in Figure S6, indicating

that the expanded parameter distributions still map to the same measurement ranges. Further, the change in parameter distributions between the second and third configurations (standard deviation equal to 1 versus 2 times the RVI value) is quite limited and most apparent in just two parameters: $\mathrm{C_{SV}}$ and $\mathrm{SBV0}$. These two parameters are highly correlated in the posterior, as will be discussed in the following section (see Figure S7), suggesting that the primary change in the less constrained prior is expanded distributions on a pair of non-identifiable parameters. As a result, there seems to be limited or no benefit to the less informative prior in terms of better capturing observable population-level variability. Therefore, we chose to move forward with the configuration where both the prior mean and standard deviation are set to each corresponding RVI parameter value, as this version balances competing demands: allowing adequate exploration of the parameter space without introducing overly wide distributions on an unidentifiable parameter pair.

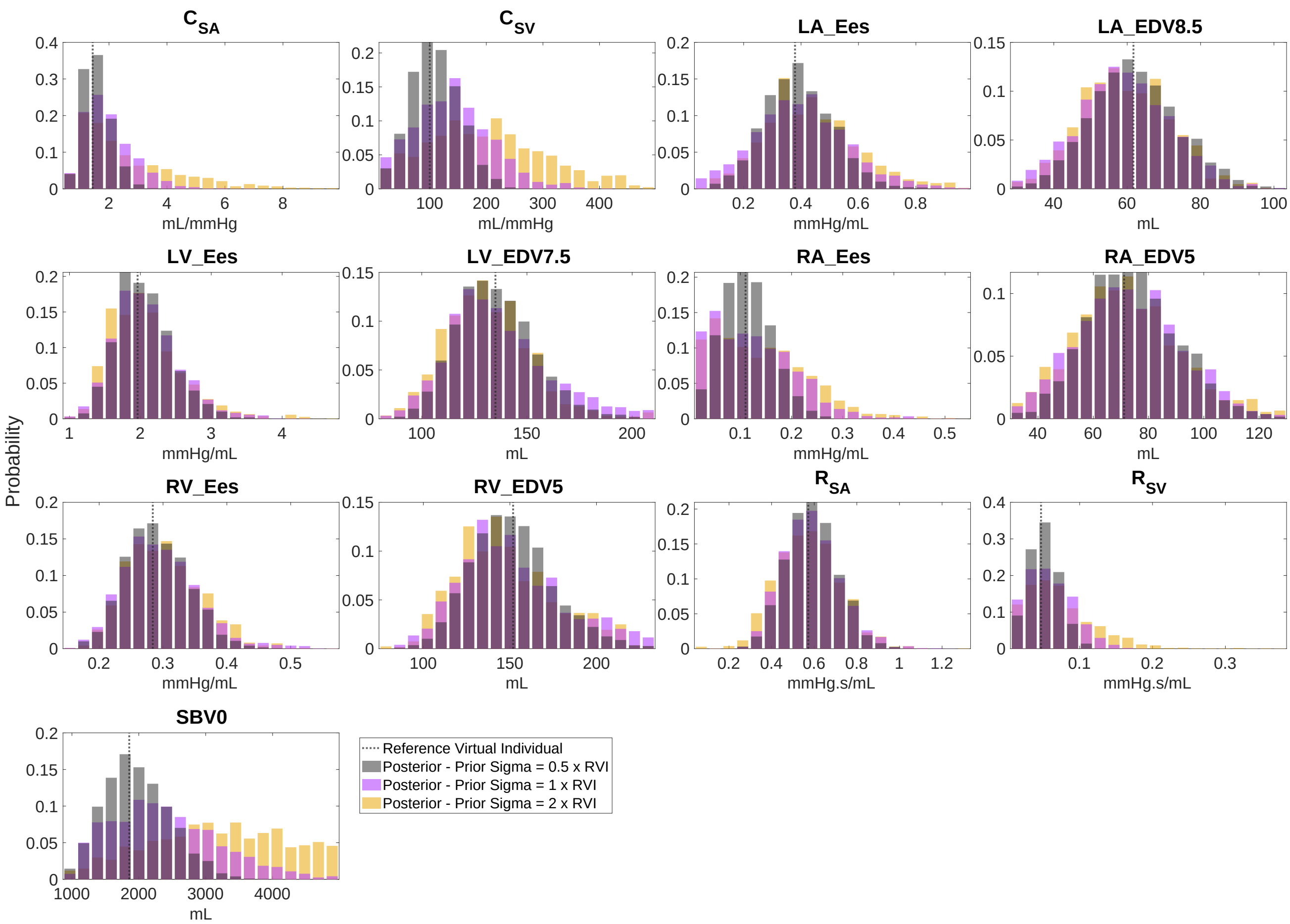

Figure S5. One-dimensional parameter marginal distributions for the population posterior obtained with three different formulations of the prior around the reference virtual individual (RVI) parameter set: prior sigma = 0.5 (grey), 1 (purple), and 2 (orange) times the RVI value.

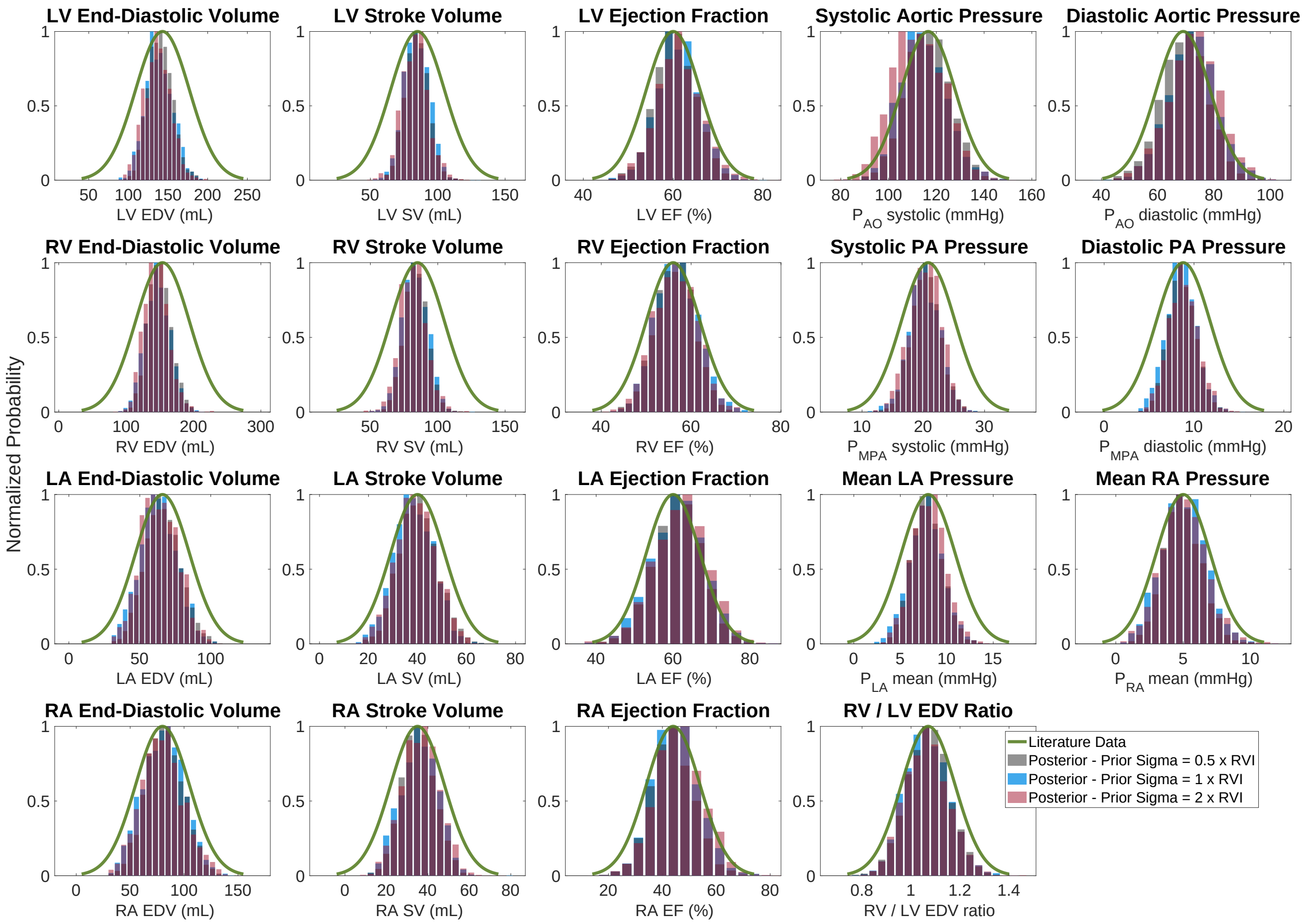

Figure S6. Model output measurement distributions corresponding to the population posterior obtained for three different formulations of the prior around the reference virtual individual (RVI) parameter set: prior sigma equal to 0.5 (grey), 1 (blue), and 2 (red) times the RVI value. Green Gaussian curves highlight the population-level measurement target data from the literature. For ease of comparison, all histograms and Gaussian curves are normalized to have a consistent peak amplitude of 1.

2.3.3 Posterior characteristics and Bayesian diagnostics

For the prior and likelihood formulation presented in the manuscript (parameter prior sigma equal to the RVI value and all 19 measurements included in the likelihood), posterior parameter correlations are depicted in Figure S7. These results highlight that many parameter pairs are non-trivially correlated within the population posterior, particularly $\mathrm{C_{SV}}$ and $\mathrm{SBV0}$. This strong positive correlation is unsurprising as $\mathrm{C_{SV}}$ is the primary contributor to overall compliance of the system, and similar simulation results can be achieved when compliance and the volume distributed throughout the system are increased proportionately. This represents a practical non-identifiability that may support future parameter space reduction by fixing one of these parameters.

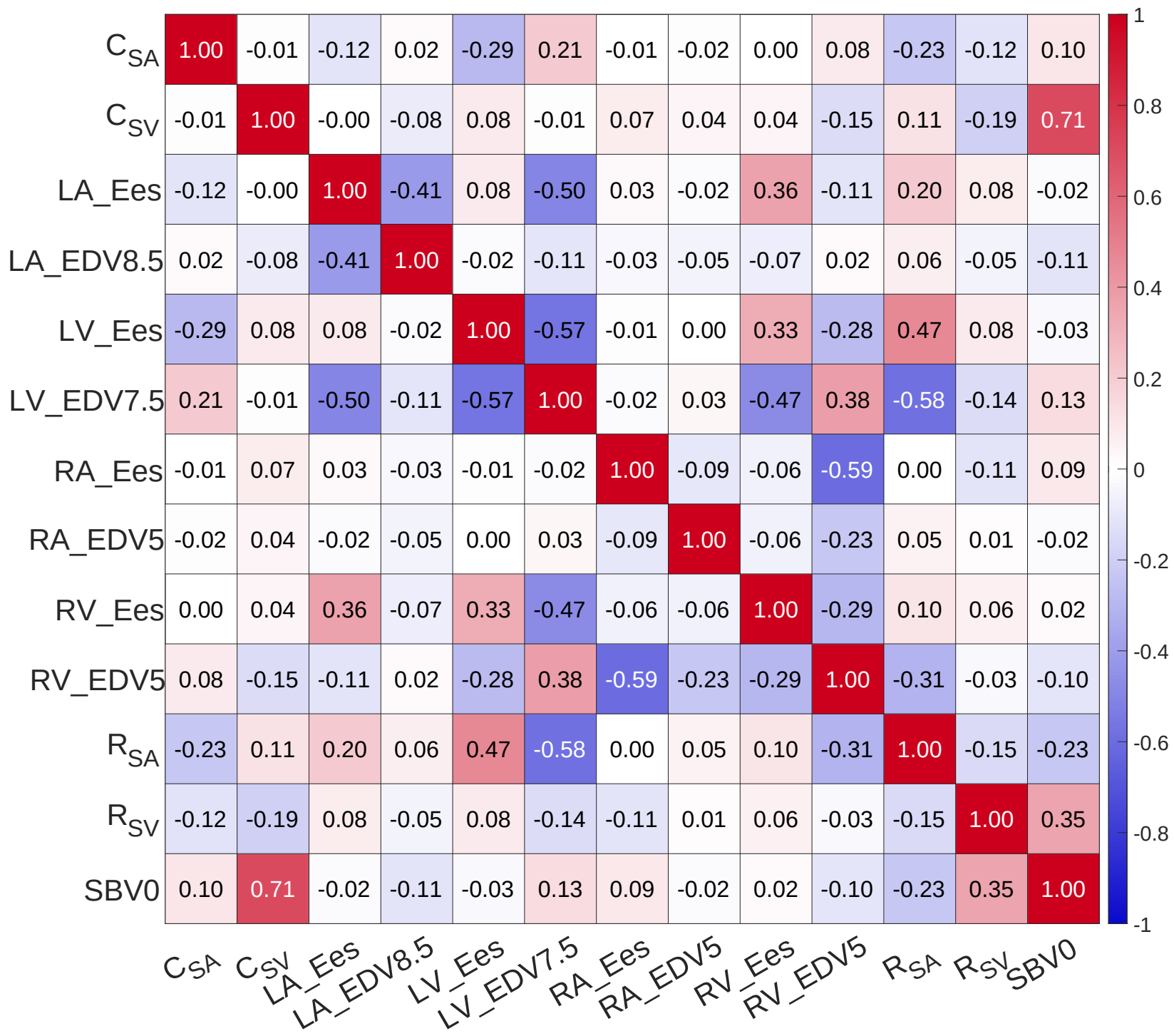

Figure S7. Posterior parameter correlation coefficients.

Additional posterior characteristics and Bayesian diagnostics are reported in Table S3, with plots of per chain parameter values, the average acceptance rate, and the multivariate and univariate Gelman-Rubin diagnostic ($\hat{R}$) values across generations provided in Figure S8. While some bulk effective sample size (ESS) values are low, stronger tail coverage suggests that the virtual population provides sufficient coverage of a range of patient phenotypes. However, this remains an area for further investigation in the context of individual patient digital twin results.

Table S3. Posterior characteristics and diagnostics for the DREAM virtual population posterior, including the posterior mean and standard deviation (SD), the Gelman-Rubin diagnostic $\hat{R}$, bulk and tail effective sample size (ESS), Monte Carlo Standard Error (MCSE), and the ratio of MCSE to the posterior standard deviation (MCSE / SD ratio) for each parameter. All values are rounded to three significant figures.

Parameter	Mean	SD	$\hat{R}$	ESS - bulk	ESS - tail	MCSE	MCSE / SD ratio
C_{SA}	1.99	0.850	1.03	226	511	0.0568	0.0668
C_{SV}	141	67.3	1.02	268	415	4.06	0.0604
LA_EDV8.5	59.1	12.3	1.05	203	317	0.863	0.0701
LA_Ees	0.402	0.158	1.02	189	272	0.0115	0.0728
LV_EDV7.5	136	23.7	1.04	108	288	2.29	0.0969
LV_Ees	2.09	0.464	1.04	175	353	0.0351	0.0758
RA_EDV5	73.0	18.5	1.01	350	430	0.986	0.0534
RA_Ees	0.126	0.0792	1.03	183	241	0.00570	0.0720
RV_EDV5	152	30.2	1.03	195	178	2.28	0.0755
RV_Ees	0.297	0.0596	1.02	337	518	0.00326	0.0548
R_{SA}	0.585	0.133	1.03	170	352	0.0105	0.0788
R_{SV}	0.0592	0.0313	1.03	245	468	0.00204	0.0653
SBV0	2390	795	1.02	238	360	50.4	0.0633

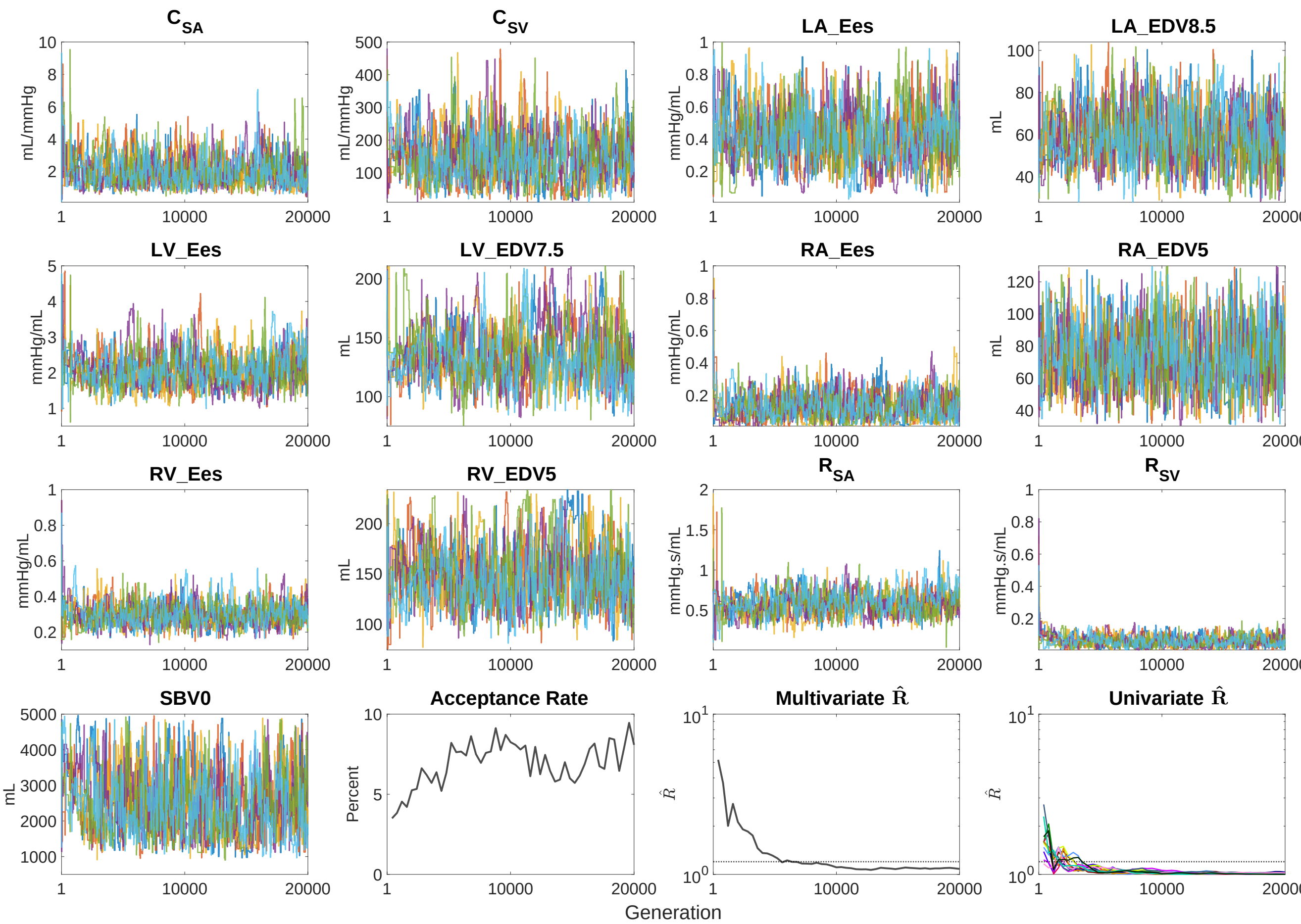

Figure S8. Parameter values per chain across generations of the DREAM algorithm (one line / color per chain), as well as the overall acceptance rate, the multivariate Gelman-Rubin diagnostic ($\hat{R}$), and the per-parameter univariate $\hat{R}$ values across generations. Dashed horizontal lines on the plots of $\hat{R}$ represent the convergence criteria (<1.2).

2.4 Patient-specific digital twins

2.4.1 MIMIC-IV patient characteristics

A summary of demographic and hemodynamic characteristics of the selected MIMIC-IV patients for whom we created patient-specific digital twins is provided in Table S4.

Table S4. Characteristics of the MIMIC-IV population. Note that weight at admission was missing for three patients and is therefore reported across only 14 patients.

Characteristic or Measurement	Count or Mean	Range
Number of patients (n)	17	n/a
Male sex (n)	15	n/a
Age (years)	50.53	31 – 68
Weight (kg)	90.14	56.1 – 130.4
Heart rate (bpm)	71.83	61 – 80
Systolic aortic pressure (mmHg)	118.47	107 – 128
Diastolic aortic pressure (mmHg)	63.58	60 – 69
Systolic PA pressure (mmHg)	20.78	18 – 24
Diastolic PA pressure (mmHg)	9.49	6 – 11.3
Central venous pressure (mmHg)	4.73	3 – 7
Cardiac output (L/min)	5.97	4.6 – 7
Computed stroke volume (mL)	83.55	66.7 – 103.3

2.4.2 Posteriors from the virtual population prior

For the patient-specific digital twins conditioned on the virtual population prior, we have provided posterior diagnostics in Table S5. The reported effective sample size (ESS) is computed according to Kish's formula (Kish, 1965). The threshold for "significant posterior mass" was set as the probability of each virtual individual (VI) in a uniform distribution (one over the total number of VIs). Mean absolute percent error (MAPE) was computed across the $M = 6$ available patient-specific measurements as follows:

$$\text{MAPE} = \frac{100}{M} \sum_{i=1}^{M} \left| \frac{\bar{y}_{twin,i} - y_{patient,i}}{y_{patient,i}} \right| \tag{S10}$$

where $\bar{y}_{twin,i}$ is the weighted mean of measurement i in the digital twin posterior distribution and $y_{patient,i}$ is the patient's corresponding clinical measurement target for the $M = 6$ patient-specific measurements. As an additional reference to demonstrate the value of the digital twin posterior over the virtual population prior, this computation was repeated using the virtual population mean in place of the digital twin posterior mean, presented as "MAPE (virtual pop.)" in Table S5, which also provides the ratio of the digital twin MAPE to the virtual population MAPE. A small value of this ratio indicates that the digital twin posterior matches the target patient much better than the virtual population prior, while a value of 1 would indicate equally good matches (in other words, no additional value of the digital twin posterior over the virtual population prior).

Table S5. Posterior characteristics for the patient-specific digital twins conditioned on the virtual population prior, including effective sample size (ESS), Shannon entropy (normalized to the entropy of an equal-size uniform distribution), the percentage of the 4.86 million total virtual individuals (VIs) which have a significant posterior mass, the mean absolute percent error (MAPE) across measurements of both the digital twin posterior mean and the virtual population prior mean for each patient, and the ratio of the twin to virtual population MAPE values.

Patient #	ESS	Normed Entropy	% VIs with sig. posterior mass	MAPE (twin)	MAPE (virtual pop.)	MAPE Ratio (twin to virtual pop.)
1	3725	0.59	1.14%	0.71%	9.18%	0.08
2	404	0.45	0.32%	2.75%	9.49%	0.29
3	1932	0.53	0.60%	2.11%	15.49%	0.14
4	241	0.44	0.38%	8.61%	24.90%	0.35
5	3420	0.58	0.87%	1.97%	19.32%	0.10
6	1266	0.51	0.45%	2.33%	16.16%	0.14
7	1118	0.53	0.80%	5.17%	8.68%	0.60
8	1594	0.55	0.69%	1.53%	8.51%	0.18
9	1099	0.51	0.55%	3.83%	19.52%	0.20
10	627	0.49	0.51%	1.87%	13.05%	0.14
11	217	0.44	0.44%	3.02%	12.62%	0.24
12	2005	0.55	0.80%	2.76%	19.74%	0.14
13	1229	0.52	0.51%	3.15%	13.11%	0.24
14	1299	0.50	0.39%	3.54%	13.36%	0.27
15	1173	0.53	0.69%	2.37%	9.57%	0.25
16	304	0.44	0.24%	2.09%	15.19%	0.14
17	1791	0.53	0.54%	1.30%	10.97%	0.12

We additionally computed the Continuous Ranked Probability Score (CRPS) for each patient and each measurement (Hersbach, 2000):

$$\text{CRPS} = \int_{\mathbb{R}} \left(F(x) - H(x - y_{patient})\right)^2 dx \tag{S11}$$

where $F(x)$ is the cumulative distribution function of the posterior, $H(x - y)$ is the Heaviside step function (0 when $x < y_{patient}$ and 1 when $x \geq y_{patient}$), $y_{patient}$ is the patient's clinical measurement target, and x represents all corresponding simulation output values of digital twin posterior. The resulting CRPS values are provided in Table S6. CRPS is reported in the units of the measurement; a value of 0 would indicate a perfect match (100% posterior probability at the target value), while larger values indicate an inaccurate and/or imprecise posterior estimate.

Table S6. Continuous Ranked Probability Score (CRPS) for each patient and each measurement (LV SV = left ventricular stroke volume, AOP = aortic pressure, PAP = pulmonary artery pressure, and RAP = right atrial pressure)

Patient #	LV SV (mL)	Systolic AOP (mmHg)	Diastolic AOP (mmHg)	Systolic PAP (mmHg)	Diastolic PAP (mmHg)	Mean RAP (mmHg)
1	1.03	0.77	0.58	0.19	0.15	0.12
2	1.66	1.71	0.65	0.30	0.10	0.22
3	1.13	0.69	0.61	0.21	0.36	0.19
4	4.80	0.71	0.68	2.52	1.46	0.09
5	1.66	0.78	0.72	0.38	0.18	0.15
6	1.14	0.83	0.74	0.30	0.31	0.13
7	3.72	0.86	0.70	1.26	0.97	0.12
8	1.12	0.94	0.76	0.39	0.23	0.10
9	2.09	1.11	0.97	0.42	0.28	0.13
10	1.33	0.79	0.83	0.23	0.28	0.10
11	1.82	0.56	0.57	0.28	0.79	0.12
12	0.97	0.75	0.55	0.31	0.18	0.21
13	2.00	0.75	0.77	0.36	0.23	0.16
14	0.98	1.17	1.32	0.21	0.91	0.24
15	1.10	0.64	0.73	0.30	0.64	0.14
16	1.56	0.55	0.58	0.46	0.27	0.14
17	1.22	1.08	0.50	0.35	0.13	0.09

2.4.3 Patient-specific DREAM digital twins

For purposes of the comparison mentioned in the Discussion of the manuscript, we also utilized the DREAM algorithm to directly obtain patient-specific posterior distributions for the four patients whose full posterior distributions are visualized in the manuscript. This analysis used the same prior configuration as described in the body of the manuscript for estimation of the population posterior, simply substituting in the patient data likelihood formulation described for obtaining patient-specific digital twins. In this way, we maintained the same parameter space and relatively uninformative prior knowledge based on population-level mean measurement values, without the further benefit of constraining the posterior on the basis of a virtual population prior.

The resulting posterior distributions show that many parameters are poorly constrained relative to the virtual population prior and the population-based posterior, as depicted in Figure S9, where the DREAM posterior in orange is often outside of both the population prior (grey) and the population-based posterior (purple). These poorly constrained parameter distributions lead in turn to poorly constrained measurement distributions for cases where patient-specific data were not available, as shown in Figure S10. Measurements with available patient-specific data (depicted by narrow solid Gaussian curves) are similarly well constrained between the virtual population-based posterior (blue) and the DREAM posterior (orange). However, other measurements without patient-specific data show that the DREAM posterior takes on values well outside of the range of a normal healthy population, plotted for reference as wide dashed Gaussian curves. For example, LV ejection fraction ranges from roughly 35% to 80% for the four patients, well outside of a normal healthy range, despite patient-specific LV stroke volumes being relatively well matched.

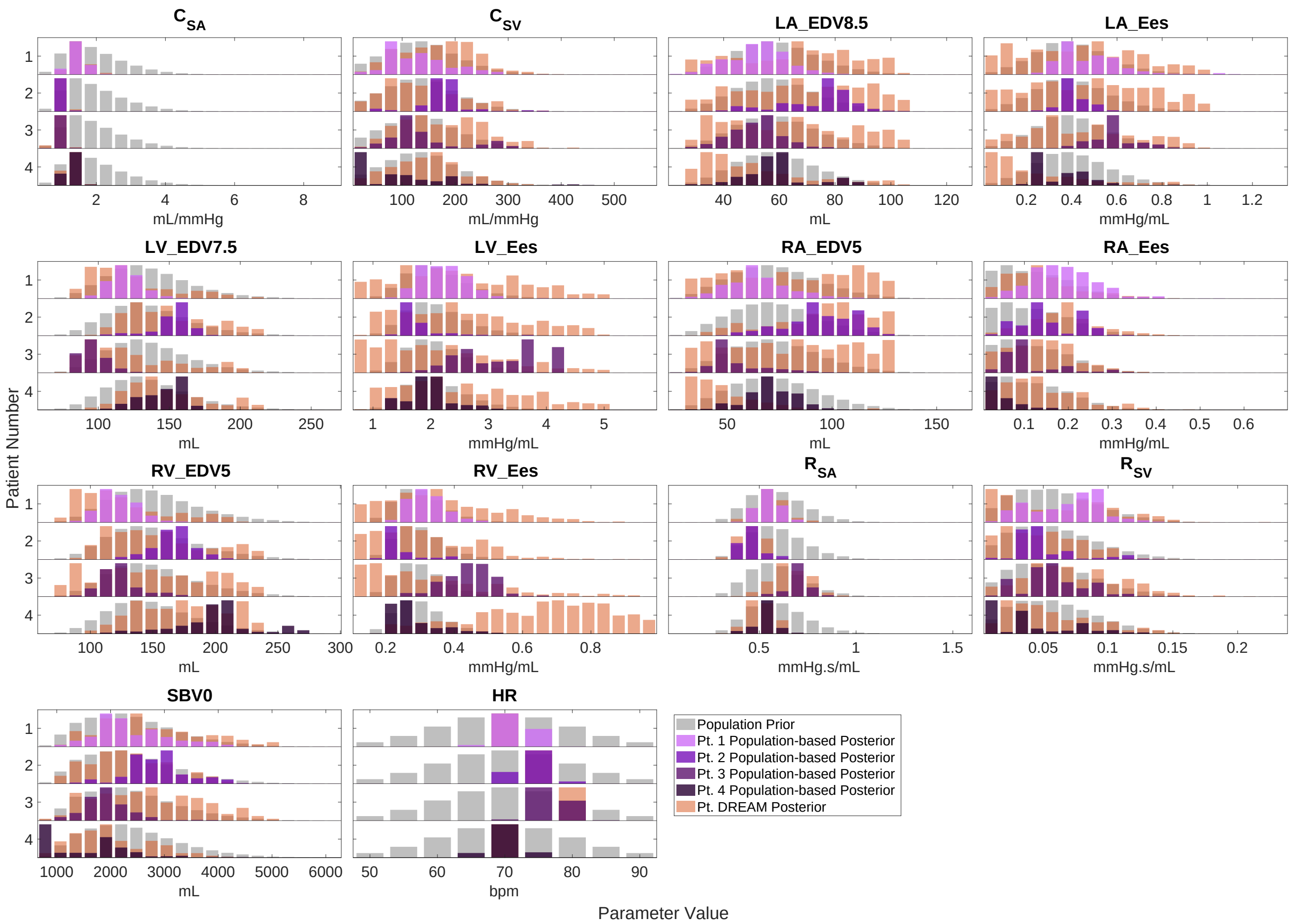

Figure S9. Posterior parameter marginal distributions for four patient digital twins in shades of purple (population-based posterior, as presented in the main body of the manuscript) and orange (DREAM posterior), with the virtual population prior distribution plotted in grey for reference. For ease of comparison, all histograms are normalized to have a consistent peak amplitude.

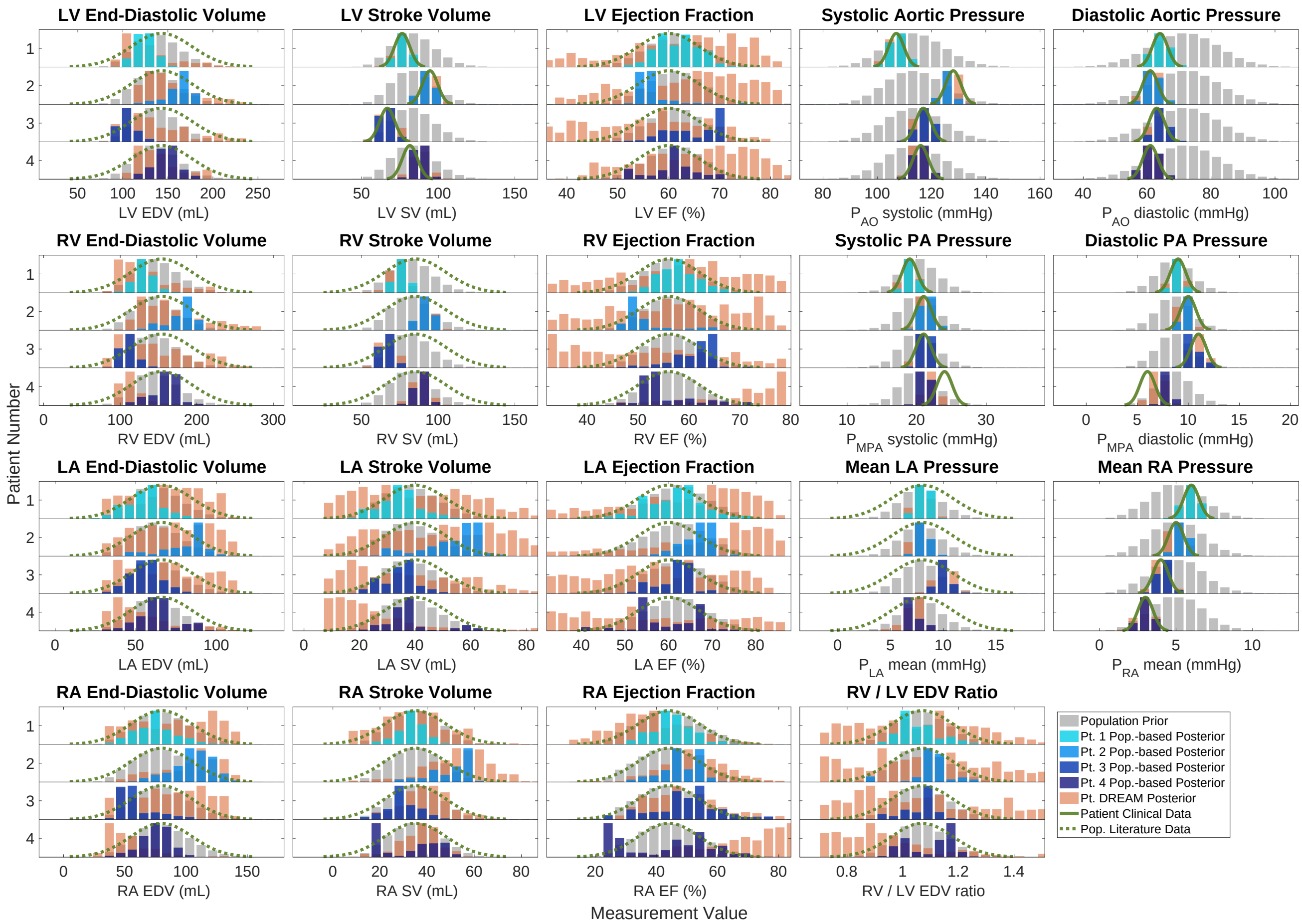

Figure S10. Model output measurement distributions corresponding to the four patient digital twin posteriors in shades of blue (population-based posterior) and orange (DREAM posterior), with the virtual population prior distributions plotted in grey for reference. For measurements with available patient-specific clinical data, the solid Gaussian curves depict the measured value and uncertainty applied in the likelihood function. For other measurements, the population-level data from the literature are plotted as a dashed Gaussian curve for reference. For ease of comparison, all histograms and Gaussian curves are normalized to have a consistent peak amplitude.

2.5 Computational details

The total offline simulation cost to arrive at the virtual population was approximately 78 hours (RVI point estimates: 9 hours, Sobol index analysis: 3.5 hours, DREAM population posterior: 11 hours, allometric scaling and varied heart rate: 54.5 hours). Note that this duration only includes the final chosen configuration at each step, for example ignoring the time required to obtain the multiple population posteriors presented in supplementary sections 2.3.1 and 2.3.2. The final virtual population of 4.86 million VIs can be stored in a MATLAB data (.mat) file of approximately 128 MB. The solver error rate amongst these simulations is less than 0.001% (42 of 4.86 million simulations).

The time to generate an individual patient posterior through DREAM can vary depending on convergence speed, acceptance rate, and other factors. For the examples presented here, this process required approximately 12 to 24 hours.

REFERENCES

Alexander Jr, J., Sunagawa, K., Chang, N., and Sagawa, K. (2018). Instantaneous pressure-volume relation of the ejecting canine left atrium. *Circulation Research* 61, 209–219. doi:10.1161/01.res.61.2.209

Bjørdalsbakke, N. L., Sturdy, J. T., Hose, D. R., and Hellevik, L. R. (2022). Parameter estimation for closed-loop lumped parameter models of the systemic circulation using synthetic data. *Mathematical Biosciences* 343, 108731. doi:10.1016/j.mbs.2021.108731

Burkhoff, D., Alexander, J., and Schipke, J. (1988). Assessment of windkessel as a model of aortic input impedance. *American Journal of Physiology-Heart and Circulatory Physiology* 255, H742–H753. doi:10.1152/ajpheart.1988.255.4.h742

Burkhoff, D. and Tyberg, J. V. (1993). Why does pulmonary venous pressure rise after onset of LV dysfunction: a theoretical analysis. *American Journal of Physiology-Heart and Circulatory Physiology* 265, H1819–H1828. doi:10.1152/ajpheart.1993.265.5.h1819

Carruthers, S., McCall, B., Cordell, B., and Wu, R. (1987). Relationships between heart rate and PR interval during physiological and pharmacological interventions. *British Journal of Clinical Pharmacology* 23, 259–265. doi:10.1111/j.1365-2125.1987.tb03043.x

Fudim, M., Kaye, D. M., Borlaug, B. A., Shah, S. J., Rich, S., Kapur, N. K., et al. (2022). Venous tone and stressed blood volume in heart failure JACC review topic of the week. *Journal of the American College of Cardiology* 79, 1858–1869. doi:10.1016/j.jacc.2022.02.050

Hersbach, H. (2000). Decomposition of the continuous ranked probability score for ensemble prediction systems. *Weather and Forecasting* 15, 559–570. doi:10.1175/1520-0434(2000)015⟨0559:dotcrp⟩2.0.co;2

[Dataset] Johnson, A., Bulgarelli, L., Pollard, T., Gow, B., Moody, B., Horng, S., et al. (2024). MIMIC-IV. doi:10.13026/kpb9-mt58. Version 3.1

Johnson, A. E. W., Bulgarelli, L., Shen, L., Gayles, A., Shammout, A., Horng, S., et al. (2023). MIMIC-IV, a freely accessible electronic health record dataset. *Scientific Data* 10, 1. doi:10.1038/s41597-022-01899-x

Kaye, D. M., Wolsk, E., Nanayakkara, S., Mariani, J., Hassager, C., Gustafsson, F., et al. (2021). Comprehensive physiological modeling provides novel insights into heart failure with preserved ejection fraction physiology. *Journal of the American Heart Association* 10, e021584. doi:10.1161/jaha.121.021584

Kish, L. (1965). Sampling organizations and groups of unequal sizes. *American sociological review* 30, 564–72

Klingensmith, M. E., Aziz, A., Bharat, A., Fox, A. C., and Porembka, M. R. (2012). *The Washington Manual of Surgery* (Wolters Kluwer Health)

Klotz, S., Hay, I., Dickstein, M. L., Yi, G.-H., Wang, J., Maurer, M. S., et al. (2006). Single-beat estimation of end-diastolic pressure-volume relationship: a novel method with potential for noninvasive application. *American Journal of Physiology-Heart and Circulatory Physiology* 291, H403–H412. doi:10.1152/ajpheart.01240.2005

Kovacs, G., Berghold, A., Scheidl, S., and Olschewski, H. (2009). Pulmonary arterial pressure during rest and exercise in healthy subjects: a systematic review. *European Respiratory Journal* 34, 888–894. doi:10.1183/09031936.00145608

Loh, W.-L. (1996). On latin hypercube sampling. *The Annals of Statistics* 24, 2058–2080. doi:10.1214/aos/1069362310

McCracken, C., Szabo, L., Abdulelah, Z. A., Vago, H., Nichols, T. E., Petersen, S. E., et al. (2023). Ventricular volume asymmetry as a novel imaging biomarker for disease discrimination and outcome prediction. *medRxiv* , 2023.11.03.23298024doi:10.1101/2023.11.03.23298024

Morissette, M. P. (1977). Colloid osmotic pressure: its measurement and clinical value. *Canadian Medical Association Journal* 116, 897–900

Petersen, S. E., Aung, N., Sanghvi, M. M., Zemrak, F., Fung, K., Paiva, J. M., et al. (2017). Reference ranges for cardiac structure and function using cardiovascular magnetic resonance (CMR) in caucasians from the UK biobank population cohort. *Journal of Cardiovascular Magnetic Resonance* 19, 18. doi:10.1186/s12968-017-0327-9

Reddy, Y. N., Obokata, M., Egbe, A., Yang, J. H., Pislaru, S., Lin, G., et al. (2019). Left atrial strain and compliance in the diagnostic evaluation of heart failure with preserved ejection fraction. *European Journal of Heart Failure* 21, 891–900. doi:10.1002/ejhf.1464

Shimizu, S., Une, D., Kawada, T., Hayama, Y., Kamiya, A., Shishido, T., et al. (2018). Lumped parameter model for hemodynamic simulation of congenital heart diseases. *The Journal of Physiological Sciences* 68, 103–111. doi:10.1007/s12576-017-0585-1

Stergiopulos, N., Meister, J. J., and Westerhof, N. (1996). Determinants of stroke volume and systolic and diastolic aortic pressure. *American Journal of Physiology-Heart and Circulatory Physiology* 270, H2050–H2059. doi:10.1152/ajpheart.1996.270.6.h2050

Suga, H., Sagawa, K., and Shoukas, A. A. (1973). Load independence of the instantaneous pressure-volume ratio of the canine left ventricle and effects of epinephrine and heart rate on the ratio. *Circulation Research* 32, 314–322. doi:10.1161/01.res.32.3.314

Vrugt, J. A. (2016). Markov chain monte carlo simulation using the DREAM software package: Theory, concepts, and MATLAB implementation. *Environmental Modelling & Software* 75, 273–316. doi:10.1016/j.envsoft.2015.08.013

Vrugt, J. A., Braak, C. J. F. t., Diks, C. G. H., Robinson, B. A., Hyman, J. M., and Higdon, D. (2009). Accelerating markov chain monte carlo simulation by differential evolution with self-adaptive randomized subspace sampling. *International Journal of Nonlinear Sciences and Numerical Simulation* 10, 273–290. doi:10.1515/ijnsns.2009.10.3.273

Westerhof, N., Lankhaar, J.-W., and Westerhof, B. E. (2009). The arterial windkessel. *Medical & Biological Engineering & Computing* 47, 131–141. doi:10.1007/s11517-008-0359-2

Wright, J. D., Hughes, J. P., Ostchega, Y., Yoon, S. S., and Nwankwo, T. (2011). Mean systolic and diastolic blood pressure in adults aged 18 and over in the united states, 2001–2008. *National Health Statistics Reports*

Zhang, X., Trame, M., Lesko, L., and Schmidt, S. (2015). Sobol sensitivity analysis: A tool to guide the development and evaluation of systems pharmacology models. *CPT: Pharmacometrics & Systems Pharmacology* 4, 69–79. doi:10.1002/psp4.6
